# A Mixed Methods Approach to Explore the Experience of Pain and Its Management in People with Parkinson's Disease

**DOI:** 10.1155/2024/8515400

**Published:** 2024-05-25

**Authors:** Vanessa Nguy, Bernadette Brady, Leanne M. Hassett, Colleen G. Canning, James M. Elliott, Natalie E. Allen

**Affiliations:** ^1^Faculty of Medicine and Health, Sydney School of Health Sciences, The University of Sydney, Camperdown, Australia; ^2^South West Sydney Local Health District, Sydney, Australia; ^3^Institute for Musculoskeletal Health, The University of Sydney, Sydney Local Health District, Camperdown, Australia; ^4^The Kolling Institute, Northern Sydney (Arabanoo) Precinct, St Leonards, Australia

## Abstract

**Introduction:**

Pain in Parkinson's disease (PD) is common but poorly understood, with most research to date taking a mechanistic approach. This mixed methods study takes a broader biopsychosocial approach to assess and describe contributors of pain and explore pain management and the relationship between pain and physical activity in people with PD (PwPD) and chronic pain.

**Methods:**

A structured survey evaluated respondents' contributors of pain using standardized, self-report assessments of the following: pain, peripheral neuropathy, central nociplastic change, emotional dysregulation or pathology, and maladaptive cognitions. Semistructured individual interviews were conducted with purposively sampled survey participants and analyzed using inductive thematic analysis.

**Results:**

Eighty-nine PwPD (mean age 67 years, 55% female) completed the survey. The most common pain contributors were maladaptive cognitions (62%), central nociplastic change (49%), and emotional dysregulation (44%). Approaches to pain management and the response to physical activity were variable within and across individuals with different pain contributors. Four themes emerged from interviews with 24 participants: (1) causative perceptions of pain are diverse; (2) sense of control influences disease acceptance and exercise self-efficacy; (3) belief in the value of therapy; and (4) pain as the unspoken PD symptom. Physical activity was used by PwPD for pain management; however, the relationship between pain and physical activity varied based on sense of control.

**Conclusions:**

Clinicians should screen for pain and assess its contributors to provide individualized, multidimensional pain management that considers the biological, psychological, and social factors of pain in PwPD. It is plausible that such an approach would promote a better sense of control for PwPD.

## 1. Introduction

Pain is “an unpleasant sensory and emotional experience associated with, or resembling that associated with, actual or potential tissue damage” [1], with chronic pain persisting beyond 3 months [2]. Studies consistently report higher prevalence of chronic pain in people with Parkinson's disease (PwPD) compared to the general population [3–5], and it is reported to be one of the most troubling impairments [6]. Despite its negative effects on quality of life, pain recognition and management are often overlooked in PwPD [7, 8].

Research on pain in Parkinson's disease (PD) has primarily focused on mechanistic approaches, investigating underlying pathophysiological pain mechanisms [3, 9, 10]. However, a more holistic approach could provide more effective pain management. The biopsychosocial model is the accepted model for understanding the pain experience [11]. Using this model, pain can be understood as an interaction between biological (including PD-related motor impairments and neurodegeneration in systems involved in pain processing and modulation [12]), psychological (e.g., attitudes, beliefs, and mood states), and social (e.g., socio-occupational and environmental) factors. Depression, anxiety, poor sleep, poor coping strategies, and disruption in social relationships are associated with worse pain in PwPD [13–15]. Despite this, research addressing biopsychosocial factors in pain management for PwPD is lacking.

Addressing the multidimensional nature of pain requires a holistic and targeted approach [16]. To facilitate this, a clinical model which creates a personalized phenotype of pain “contributors” has been developed [17]. While not exhaustive, biopsychosocial contributing factors to pain include nociceptive input, emotional dysregulation (e.g., anxiety and depression), maladaptive cognitions (e.g., pain-related worrying), and socioenvironmental context. Identifying an individual's contributors may better equip healthcare providers to provide more individualized and informed clinical assessment and management.

To personalize pain management for PwPD, evidence is needed to guide effective approaches [18]. One recommended strategy is physical activity (i.e., bodily movement produced by skeletal muscles that requires energy expenditure [19]) including exercise (i.e., a type of planned, structured, repetitive, and purposeful physical activity [19]). Regular exercise is recommended for PwPD given its benefits (i.e., improved mobility, balance and strength, reduced falls, and possible slowing of disease progression) [20–23]. While exercise is recommended for people with chronic pain [24], its effect among PwPD remains uncertain. Theoretical evidence suggests carefully designed exercise may reduce pain by promoting positive neuroplastic and neurochemical changes [25], with some trials showing pain reduction [26]. Recent work found exercise-induced hypoalgesia (reduction in pain sensitivity) in most PwPD following exercise [27]. However, while group results were encouraging, some PwPD did not experience exercise-induced hypoalgesia. Unexpectedly, further work found an association between increased physical activity and worse pain among PwPD [13]. These results highlight the need to better understand the relationship between exercise and pain.

We hypothesized that identifying the contributing factors of pain experienced by PwPD will reveal valuable insights into developing effective, holistic, and individualized pain management interventions. Therefore, this study aimed to (1) assess and describe the primary contributors of pain, (2) explore similarities and differences in approaches to pain management among people with different contributors, and (3) describe the relationship between pain and physical activity among people with different pain contributors.

## 2. Methods

### 2.1. Design

A mixed methods study utilizing an explanatory sequential design was undertaken from April to December 2021, using qualitative data (semistructured interviews) to support the interpretation of quantitative (survey) results (Supplementary Figure (available here)). This design was chosen to accommodate a more robust analysis than either approach alone, adding contextualizing information to comprehensively understand pain management techniques choices and their relationship with physical activity [28]. The choice of a mixed methods approach was underpinned by an ontology of pragmatism, accepting that there can be single or multiple realities to a phenomenon, and these can be accessed via a range of methods and flexible approaches [29]. Specific to the qualitative phase, an epistemology of interpretivism guided the analysis, prioritising understanding subjective interpretations of experiences [30]. Survey participants consented by submitting the survey and interview participants provided written informed consent. This report follows the COREQ-32 [31] checklist for qualitative studies and the GRAMMS [32] checklist for mixed methods. Ethics approval was obtained from the University of Sydney Human Research Ethics Committee (project number 2021/035).

### 2.2. Study Participants

Participants were recruited across Australia through the following: the research team's database of PwPD who had previously agreed to be contacted regarding research participation; written and online advertisements in publications aimed at PwPD; and health professionals/clinics advertising the survey within local networks. Volunteers were included if they had idiopathic PD diagnosed by a neurologist and chronic pain (self-reported pain intensity ≥3/10 for ≥2 days/week in the prior 3 months). Volunteers were excluded if they had previous spinal surgery or whiplash, a fibromyalgia diagnosis, or were unable complete the survey due to insufficient cognitive or English language ability. Additionally, participants selected for interviews were screened over the phone for substantial cognitive impairment and excluded if they scored <5 on the Memory Impairment Screen [33]. Purposive sampling from the survey ensured diversity in gender, disease duration, PD severity, and main pain contributor among interviewees. Recruitment continued until saturation of concepts was achieved across interviews [34].

### 2.3. Data Collection and Measures

#### 2.3.1. Survey

Survey data were collected online (via Qualtrics) or on paper, based on participant preference. The survey (Supplementary material (available here)) captured background information including age, gender, PD duration, PD severity (self-rated using the Hoehn and Yahr Scale [35]), PD medication, pain severity, pain therapy, physical activity levels, and response of pain to physical activity. Physical activity levels were assessed through a specific question in the survey, where participants were asked to report the number of days in the past week they engaged in physical activity lasting at least 30 minutes, which was sufficient to increase their breathing rate. This question aligns with the general recommendation for older adults of 30 minutes of moderate-intensity physical activity per day [36].

The survey also included global pain measures (the King's PD Pain Questionnaire [37] and Brief Pain Inventory (BPI) [38] severity and interference subscores) and measures of potential pain contributors. Five contributors were selected to summarize different domains of a person's pain experience that were assessable through self-report questionnaires: (1) peripheral neuropathy (Self-reported Leeds Assessment of Neuropathic Symptoms and Signs pain scale (S-LANNS) [39]), (2) central nociplastic change (Central Sensitization Inventory (CSI) [40]), (3) emotional dysregulation or pathology (Parkinson's Anxiety Scale (PAS) [41]), (4) maladaptive cognitions (Pain Catastrophizing Scale (PCS) [42] and Pictorial Fear of Activity Scale (PFACTS) [43]), and (5) socioenvironmental context (median income and employment status). Definitions, measurement tool(s), and tool(s) descriptions for each contributing factor can be found in Table 1 (brief version) and Supplementary Table 1 (expanded version).

#### 2.3.2. Interviews

Individual semistructured interviews were conducted by the first author (VN) via Zoom videoconferencing (Zoom Video Communications Inc, San Jose, CA, USA) within 2 months of survey submission. Participants knew the researcher was a female exercise physiologist completing a PhD but had no prior relationship with her. The interviewer was supported by an experienced qualitative researcher (BB) to upskill in interviewing, including the completion of practice interviews. An interview guide [44] was developed based on similar research in other populations [45], piloted with a consumer with chronic pain (without PD), and subsequently refined (Supplementary material (available here)). The guide was designed to explore different aspects of the pain experience broadly (i.e., physical, social, and emotional), pain management approaches, and relationships between physical activity and pain, while also allowing for flexibility to explore meaning and allow divergence into relevant topics as they arose. Interviews were digitally audio-recorded and transcribed verbatim. Field notes and reflections were made after each interview to record contextual information and contribute to reflexivity. Transcripts were not returned to participants for comment or correction in order to avoid overburdening them [46]. However, during the interview the main points were regularly summarized to allow participants to clarify or comment on them.

### 2.4. Data Analysis

#### 2.4.1. Survey Analysis

Quantitative data were analyzed using the Statistical Package for the Social Science (version 26; IBM SPSS statistics). Each questionnaire was scored and cutoffs were used to categorize pain contributors as very low, low, moderate, high, and very high (Supplementary Table 1). The two maladaptive cognition measures were categorized independently, and the average categorization from both measures determined this factor's category. Main pain contributors were those categorized the highest for that participant; therefore, participants could have more than one main pain contributor. Descriptive statistics were calculated for participant characteristics and pain contributors.

#### 2.4.2. Interview Analysis

Inductive thematic analysis was conducted using NVivo 12 (QSR International) by a research team comprised of an experienced qualitative researcher (BB), investigators with qualitative coding experience (NA, LH, and CC), one involved in qualitative research more generally (JE), and one who attended coursework training for their PhD (VN). The team comprised of five physiotherapists (BB, NA, CC, LH, and JE) and one exercise physiologist (VN), who have experience working with people with chronic pain and/or Parkinson's disease. As part of familiarization, transcripts were read multiple times by the primary author (VN). Open coding was conducted independently by three researchers (VN, BB, and NA) on four transcripts to create a preliminary codebook and examined over several meetings ensuring critical data were not omitted and to offer alternative views [47]. VN then coded successive transcripts as they were conducted, adding new codes as required, regularly meeting with a second researcher (BB) who examined the transcripts, reviewed the coding, and discussed reflective memos. A further sample (15%) of transcripts was double-coded by the broader research team using the codebook. The process of constructing, revising, and defining preliminary themes was conducted at length by the primary author (VN) in collaboration with BB and NA. Final themes were discussed and agreed upon by all authors.

Integration of qualitative and quantitative results was performed using a series of comparative matrixes created in Excel following the procedures outlined by Guetterman and James [48]. Each matrix explored patterns for the qualitative themes across different pain contributors, participant characteristics, and pain management approaches.

## 3. Results

### 3.1. Participants

Eighty-nine participants completed the survey and 24 were interviewed, with all participants invited to an interview accepting. Interviews averaged 43 minutes (range 21 to 67 minutes). Thirty-two volunteers were excluded from the survey: 4 did not have idiopathic PD diagnosed by a neurologist, 13 did not meet the criteria for chronic pain, 12 had a history of spinal surgery, and 3 had a previous diagnosis of whiplash.  Table 2 summarizes participants' demographic characteristics and Table 3 summarizes the pain contributor data. The interviewed subgroup had very similar demographic and pain categorization to the overall sample.

Overall, participants were an average of 67 (SD 9) years old, with even gender distribution (women 55%). The average time since PD diagnosis was 8 years (SD 7) and most had mild to moderate disease (Hoehn and Yahr stage 1–3). Nearly all reported musculoskeletal pain (*n* = 81, 91%), dyskinetic pain (*n* = 77, 87%), and/or fluctuation pain (*n* = 72, 81%) using the King's PD Pain Questionnaire. The mean BPI pain severity score was 4.6/10 (SD 1.7) and pain interference score was 5.5/10 (SD 2.2), indicating moderate pain severity and interference. The average participation in physical activity (including exercise) was 4.5 days per week. The reported effect of physical activity on pain varied from a large increase in pain to complete pain relief. Over half the sample reported using some form of pain therapy such as pain medication, heat, and allied health professionals (Table 4 for a complete list) with the average pain relief being 52% reduction.

Participants reported an average of two main pain contributors, determined by the contributor(s) marked highest on the categorization (range 1 to 4). The most common was maladaptive cognitions (overall *n* = 56, 62%; interviewed *n* = 19, 79%), followed by central nociplastic change (overall *n* = 44, 49%; interviewed *n* = 11, 46%), emotional dysregulation/pathology (overall *n* = 39, 44%; interviewed *n* = 5, 21%), and then peripheral neuropathy (overall *n* = 22, 24%; interviewed *n* = 7, 29%). We were unable to interpret the socioeconomic status contributor with the data we collected, in part due to a large proportion of retirees.

### 3.2. Mixed Methods Integration

Our comparative analysis explored patterns in pain management approaches related to different pain contributors within the qualitative data. No patterns emerged that characterized the pain management approaches adopted by those with similar pain contributors. Rather, there was variation in the pain management strategies amongst participants who shared a pain contributor and across those with different contributors (Table 4). Similarly, the comparative matrix did not reveal specific patterns between the quantitative components of age, gender, years since diagnosis, PD severity, days of physical activity, effect of physical activity on pain, and pain contributors with the qualitative themes.

The thematic analysis results suggested other factors characterized participants' pain management choices and experience and their relationship with physical activity. The four emergent themes were (1) causative perceptions of pain are diverse, (2) sense of control, (3) belief in the value of therapy, and (4) pain as the unspoken symptom of PD. Quotes supporting each are displayed in Supplementary Table 2.

#### 3.2.1. Theme 1: Causative Perceptions of Pain Are Diverse

Despite everyone experiencing chronic pain and PD, there were diverse perspectives regarding causation along the spectrum that pain and PD were intertwined through to pain and PD being unrelated diagnoses. Causative beliefs were shaped by (i) the timing, onset, and co-occurrence of symptoms, (ii) experiences with therapies, and (iii) experiences with healthcare professionals. Importantly, a participant's primary perception of causation uncovered insight into their pain therapy choices, with those who perceived pain as PD-related more commonly preferencing therapies that are specifically designed or targeted towards PwPD or resigning themselves that “*this chronic degenerative disease is only gonna get worse*” (P10, PD-2 yrs).

The timing, onset, and co-occurrence of symptoms were considered a major contributor to participants' perspectives that pain and PD were or were not related. For many, motor impairments such as rigidity and dystonia were considered causative or associated with pain. In contrast, participants with an injury history or pain diagnoses before the diagnosis of PD were more likely to attribute their pain to other causes, such as “*a preexisting condition…that deteriorated*” (P13, PD-6 yrs), “*a slipped disc…aggravated a bit more*” (P5, PD-5 yrs) or ageing where “*I've just worn myself out*” (P15, PD-1 yr). Along this spectrum were participants who communicated the distinction between the two was less causative but rather had an interaction effect whereby “*since Parkinson's all of these others [pain] seem to have amplified [and] become more bothersome*” (P9, PD-3 yrs) and preexisting symptoms such as “*pain…before I had Parkinson's diagnosed…[have] been far worse with Parkinson's*” (P7, PD-16 yrs). Lack of diagnostic certainty was prominent and challenging for some who were left to try to make sense of the relationship.

For the cohort, causative perceptions were shaped and consolidated by therapeutic experiences. Specifically, a reduction in pain to PD-specific procedures where “*the pain used to be a lot worse, before I had DBS [deep brain stimulation]*” (P1, PD-17 yrs) or the administration of dopaminergic drugs where the pain was “*alleviated by the levodopa [PD medication], and when that wears off it comes back*” (P1, PD-17 yrs) reinforced a participant's belief pain was linked with PD. For others, positive effects of therapy targeting diagnosed pain type, such as anti-inflammatory medications, established firm beliefs about pain being distinct from their PD because “*that's [pain] all gone because of the medication I'm on so it appears to be that that was rheumatoid arthritis*” (P15, PD-1 yr).

Lastly, healthcare provider opinions played a role in shaping or challenging participants' perceptions of the causation of pain. Participants who believed their pain was independent of PD were commonly informed by a medical provider their pain stemmed from another diagnosed pain condition/injury. Similarly, for those who already perceived PD and pain were linked, opinions that “*it's your Parkinson's…getting in the way*” (P14, PD-17 yrs) served to further consolidate this association, reflecting an inherent trust in the medical profession held by many participants. While views between participants and healthcare professionals aligned for many, this was not universal, with some participants challenged by the healthcare professional opinions that “*Parkinson's hasn't got pain associated*” (P17, PD-14 yrs), despite a participant's experience of co-occurrence of pain with symptoms of stiffness, slow movement, poor concentration, and cramps.

#### 3.2.2. Theme 2: Sense of Control

This theme characterizes how much control participants perceived they had over their pain and PD; that is, to what extent they believed that their actions or attitudes could influence their perceived pain. This theme emerged along a spectrum of responses in which some felt they had little control over their pain while others perceived great control.

Within this theme, patterns of perceptions within the spectrum became obvious. Participants who communicated a higher sense of control used language and descriptors that suggested a level of acceptance of their condition, therapy, and general situation. This acceptance often flowed into remarks about accountability, proactivity, and action-oriented coping strategies. Accompanying this sense of accountability was a high level of engagement with exercise and pain management, and greater independence in how they approached this. Even among those who did not derive pain-relieving benefits from exercise, participants cited that exercise was “*not a lost cause…there's always some benefit to be gained even if it's only short term*” (P13, PD-6 yrs) and reflected on the broader merits such as “*helping me physically,…mentally and making me realize that I can do normal things and I can be like a normal person*” (P6, PD-5 yrs). When it came to managing their exercise, these participants also communicated a higher degree of self-efficacy and control regarding their ability to exercise. This sense of control was defined by their ability to self-manage and monitor their exercise routine utilizing pacing and adapting strategies, even given constraints and impediments such as pain.

In contrast, participants who communicated a lower sense of control appeared to be preoccupied with the unhelpful or unpleasant aspects of their diagnosis and pain situation. This perceived uncontrollability of pain was often accompanied by avoidance-oriented strategies where pain dictated behavior. Among these participants, symptoms were often attributed to external factors such as apathy, depression, and disease progression. Participants expressed more pervasive feelings of anxiety and depression where “it's always there in the back of your mind and you're always trying to think of ways to minimize it and it just means that your tolerance for anything else is much reduced because you spend most of your mental time dealing with the pain” (P21, PD-22 yrs). There was also discontentment with their current therapy modalities and participants described searching for alternatives which “*all help but it only helps the symptoms temporarily and doesn't help the underlying issues*” (P11, PD-12 yrs). When it came to exercise and physical activity, these participants communicated low exercise self-efficacy and described a greater number of barriers to physical activity participation. Despite acknowledging that exercise overall was beneficial, the lack of immediate benefits contributed to a lack of motivation to adhere to reap the rewards.

While two main patterns of control were identified, there was a spectrum with some participants communicating less extreme senses of control. Among these participants, less trepidation and pessimism about their situation were communicated, though they were also less insightful and still retained some externalized onus of control for their pain. They perceived that they had some influence over their situation, but simultaneously communicated that they were lacking in certain skills, such as pacing, modification of activities, and acceptance of physical limitations. These participants used language and descriptors like “*having to learn,*” “*trying,*” and “*getting better at,*” which communicated their desires to improve.

#### 3.2.3. Theme 3: Belief in the Value of Therapy

This theme describes the beliefs that participants had about how different pain-specific therapies affect their pain. Participants reported engaging in a variety of therapies, including physical activity, medications, and physical and mindfulness therapies. For some therapies such as over-the-counter oral analgesics and physical (e.g., massage, heat) and mindfulness therapies, these strategies were trialed by participants regardless of their perceptions of pain and sense of control; however, discontinuation occurred when the intended therapeutic effect was not achieved. In such cases, these strategies appeared to offer short-term relief and provide comfort when in pain but did not provide a long-lasting solution. However, for exercise therapy, a consistent pattern emerged between a participant's causative perceptions of pain (theme 1) and sense of control (theme 2). Specifically, a participant's sense of control influenced their decisions to engage with exercise as a form of therapy, with greater use among those with a higher sense of control and lower engagement among those with low control.

Among these approaches, a notable emphasis on beliefs emerged, as participants held strong beliefs in the positive benefits of exercise for managing their PD. Most participants believed “*moving and exercise are essential*” (P9, PD-3 yrs) and “*exercise is one of the best things for Parkinson's*” (P18, PD-17 yrs), reflecting the perception that exercise could delay PD progression and facilitate mobility, independence, and function. Some participants who engaged in exercise communicated that “*it's more about just being fit, healthy, keeping mobile, as opposed to reducing pain so I can have mobility—there's a subtle difference there*” (P3, PD-2 yrs). Alongside PD-specific benefits, other participants communicated a belief that exercise reduces their pain and “*can't imagine trying to manage the Parkinson's pain or any pain without exercise*” (P20, PD-3 yrs).

Those who had strong beliefs and felt positively about exercise maintained this positivity and were less resistant to setbacks even if an exercise or activity exacerbated their pain. Rather than stopping, they adapted and described reducing or modifying, or swapping to a different exercise whereby “*if I didn't have the pain I probably would do a little bit more exercise, certainly I would do a bit more walking*” (P2, PD-5 yrs) and “*it reduces the things that I can do*” (P20, PD-3 yrs).

Participants' beliefs on perceived cost-benefit trade-off impacted decisions to engage with physical activity, where they decide whether an amount of effort (physical activity) is “*worth it*” for the “*reward.*” There were many indirect rewards associated with physical activity: joy, stimulation, distraction, physical and mental effects, and social opportunities. Participants often applied a similar philosophy of beliefs to medications and other therapies, where they described weighing up perceived efficacy and risk of therapies. Where participants experienced positive effects with certain medications but had concerns (such as side effects and addiction), they were less likely to regularly engage with medication and only use it if “*worse comes to worse*” (P13, PD-6 yrs). Where medications did not have the intended therapeutic effect, participants would disengage citing that they were “*not gonna take [the medication]…if it's not doing anything and no harm is being done, so I didn't take the medication*” (P9, PD-3 yrs). Similarly, participants perceived non-exercise and non-medicinal therapies such as physical (e.g., massage and heat) and cognitive (e.g., mindfulness) coping strategies as less risky and communicated less reluctance to trial these therapies, but also discontinued if they did not have the intended therapeutic effect. This was important because it revealed that physical activity, specifically exercise, is perceived by participants as a tool or modality to alter their disease experience.

#### 3.2.4. Theme 4: Pain Is the Unspoken Symptom of Parkinson's Disease

This theme relates to the impact of living with pain and PD. Participants communicated that living with both PD and pain “*kind of makes your world get smaller and more sort of limited*” (P1, PD-17 yrs) exacerbating feelings of isolation and leading to reduced social participation. The experience of pain was not well understood by others, further contributing to feelings of isolation and negatively impacting mood and functioning, leading to social withdrawal because “*the thing about pain is you really can't share it…you can share joy…we can share all those social actions with other people. Pain is not, pain is totally yours*” (P20, PD-3 yrs).

Participants felt healthcare providers involved in the management of their PD did not address their pain. This created feelings of frustration with medical providers, as participants perceived them as being unhelpful, dismissive, and lacking understanding when it came to addressing and identifying pain, with the focus often being on short-term relief rather than long-term management. Regardless, participants expressed low expectations of their medical professionals as they believed their “*hands were tied*” (P1, PD-17 yrs) and there was “*nothing we can do about it and that's okay but by treating it secondary to my other Parkinson's symptoms I don't think they're giving it enough attention*” (P21, PD-22 yrs).

Participants who engaged in exercise sessions with allied health professionals received support with managing their PD impairments and activity limitations including balance, mobility, gait, and everyday activities. However, their pain was not addressed unless experienced during a session. Participants reflected their allied health professionals had a reactive approach in these situations, focusing on modifying exercises and addressing the pain at that point in time. They felt this approach was narrow and did not consider pain as part of their disease burden.

## 4. Discussion

This mixed methods study highlights how thoughts and beliefs regarding pain causality and sense of control influence chronic pain experience and management in PwPD. Results demonstrate multiple contributors, with maladaptive cognitions, central nociplastic change, and emotional dysregulation/pathology being most common. Mixed methods integration failed to find patterns in pain management related to the pain contributors, with management approaches and their success varying greatly. However, patterns within the qualitative data indicated that causative perceptions of pain and sense of control influenced beliefs about the value of therapy. PwPD with greater sense of control were more accepting of their circumstances, more successful in managing their pain, and proactive in exercise engagement. Overwhelmingly, participants described their pain as being PD's unspoken symptom, receiving little attention from healthcare professionals and causing social isolation.

This study reinforces previous findings that psychological domains influence the pain experience in PwPD [14, 16, 49, 50]. Aligning with this, maladaptive cognitions, emotional dysregulation/pathology, beliefs, and sense of control were identified as important influencers. This suggests active coping strategies such as cognitive-behavioral therapy and mindfulness may be warranted. However, to our knowledge there are no clinical trials exploring psychological interventions' effect on pain in PwPD. This might explain why none of the participants engaged with psychiatrists or psychologists in their pain management in this or previous studies [16].

Pain significantly impacts quality of life and requires active management [50]. However, our study concurred with previous results that healthcare providers typically did not assess or address pain [49]. Alarmingly, few participants involved their medical (2%) and allied health professionals (22%) in their pain management, lower than previously reported [16, 51]. Nonetheless, some participants reported that healthcare providers influenced their perceptions about the cause of their pain, and this in turn affected beliefs about pain management. Furthermore, many had multiple contributors of pain and participants' sense of control tended to influence their ability to manage pain. These findings highlight the need for healthcare providers to recognise and assess pain and its potential biopsychosocial contributors, refer to multidisciplinary team members, and collaborate with PwPD for tailored and individualized long-term treatment plans.

Physical activity could be an integral part of pain management in PwPD as it potentially affects all biopsychosocial model factors [52]. It can improve physical and psychological wellbeing, promote active coping skills, and reduce disability, social isolation, anxiety, and depression [20, 23, 53]. However, research on the efficacy of physical activity-based interventions for pain in PwPD is lacking and only 12% of participants considered it as a pain management strategy. Yet, given its importance for the overall PD management [23, 53], all PwPD should ideally be referred to an exercise specialist (physiotherapist or exercise physiologist) soon after diagnosis [54]. Therefore, these specialists should regularly screen for pain and its biopsychosocial contributors and develop individualized exercise programs that address and monitor pain on a patient-by-patient basis.

Several limitations should be considered when interpreting these findings. While our study collected socioenvironmental data, the fact that most participants were retired and lack of comprehensive socioeconomic data made it difficult to draw meaningful conclusions. Nevertheless, socioenvironmental factors may impact PwPD's access to appropriate healthcare providers for pain management. Ethnicity and culture variables were not explored, yet we acknowledge their potential influence on the data. Additionally, our study only evaluated current pain management strategies without gathering information on previous strategies, which may have affected our understanding of PwPD's pain experience. Moreover, relying solely on questionnaires to assess pain contributors limited our ability to identify patterns, as in-person testing was not conducted. In addition, the study relied on participants to self-report their Parkinson's disease diagnosis and disease severity and we did not ask for detailed information about PD medications. While populations such as those with spinal surgery, whiplash, and fibromyalgia, known to influence pain sensitivity and impair pain inhibition [55–57], were excluded, individuals with other conditions possibly related to pain, such as diabetes and oncologic disorders, were not excluded. The reliance on self-report measures, absence of detailed information about PD medications, and inclusion of individuals experiencing pain due to conditions such as diabetes and oncologic disorders may have potentially influenced the study outcomes.

## 5. Conclusion

Pain in PwPD is a complex phenomenon requiring a comprehensive biopsychosocial approach to identify and address the varied biological, psychological, and socioenvironmental contributors to a person's pain experience. A multidisciplinary approach involving psychologists and exercise specialists warrants further research towards improving the assessment and management of pain in PwPD.

## Figures and Tables

**Table 1:** Description of measurements (brief version).

Measurement tools used	Description
*Global pain measures*	
The category(s) of pain present, along with the severity of pain and its level of interference on daily activities	
The King's PD Pain Questionnaire^*∗*^	(i) Time frame: past month (ii) Identifies categories of pain (iii) 14-item scale, consisting of 7 domains: musculoskeletal, chronic, fluctuation related, nocturnal, oro-facial, discoloration and edema/swelling, radicular pain (iv) Score range: 0–14
Brief Pain Inventory-severity and interference subscores^*∗*^	(i) Time frame: past week (ii) 8-item questionnaire (iii) Pain severity and interference subscores (iv) Subscore range: 0–10

*Peripheral neuropathic contributor*	
Pain caused by a lesion or disease of the peripheral nervous system	
Self-reported Leeds Assessment of Neuropathic Symptoms and Signs pain scale (S-LANNS)^*∗*^	(i) Time frame: past week (ii) 7-item scale (iii) A score ≥12 suggests pain of predominantly neuropathic origin (iv) Score range: 0–24

*Central nociplastic contributor*	
Pain that arises from altered nociception despite no clear evidence of actual or threatened tissue damage causing the activation of peripheral nociceptors or evidence for disease or lesion of the somatosensory system causing the pain	
Central Sensitization Inventory (CSI)^*∗*^	(i) Time frame: time period not specified (ii) 25-item assessment (iii) A score ≥40 is suggestive of central sensitization syndrome (iv) Score range: 0–100

E*motional dysregulation or pathology contributor*	
Impaired ability to regulate and or tolerate negative emotional states, including anxiety	
Parkinson's Anxiety Scale (PAS)^*∗*^	(i) Time frame: past month (ii) 12-item scale with three subscales, for persistent, episodic anxiety, and avoidance behavior (iii) A score of 13/14 is suggestive of patients with anxiety disorder (iv) Score range: 0–48

*Maladaptive cognitions contributor*	
Inaccurate or irrational beliefs, thoughts, or behaviors about, or resulting from, the experience of pain	
Brief Pain Catastrophizing Scale (PCS)^*∗*^	(i) Time frame: time period not specified (ii) 4-item scale (iii) A score of 6 and 9 is suggestive of a moderate and high level of catastrophizing (iv) Score range: 0–16
Pictorial fear of Activity Scale (PFACTS)^*∗*^	(i) Time frame: time period not specified (ii) Instrument with 19 images of a model engaged in a variety of activities (iii) Score range: 0–190

*Socioenvironmental contributor*	
Socioeconomic factors (e.g., income and employment status) which can interact with physical pathology to modulate a patient's report of symptoms and may worsen the clinical presentation but also access to appropriate care	
Median income ($AU)	(i) Time frame: yearly household income assessed before tax
Employment status	(i) Time frame: current employment status (i.e., home duties, retired (self-funded), retired (pensioners), currently unemployed, working part time, and working full time)

^*∗*^High score is worse.

**Table 2:** Demographic characteristics.

	Whole sample *n* = 89		Interviewed sample *n* = 24	
	Mean (SD) or number (%)	Range	Mean (SD) or number (%)	Range
Age (years)	66.5 (9.4)	35–87	66.6 (8.8)	40–79

Gender	Male: 38 (42.7%) Female: 49 (55.1%) Other: 2 (2.2%)		Male: 12 (50%) Female: 12 (50%)	

Time since diagnosis (years)	8.0 (7.1)	0–42	7.8 (6.5)	1–22

Medication for PD	Yes: 87 (97.8%) No: 2 (2.2%)		Yes: 24 (100%)	

Living situation	On your own: 15 (16.9%) Spouse or partner: 69 (77.5%) Carer: 2 (2.2%) Aged care residence or facility: 2 (2.2%) Other: 1 (1.1%)		On your own: 7 (29.2%) Spouse or partner: 16 (66.7%) Carer: 1 (4.2%)	

PD severity (self-reported Hoehn and Yahr stage)	Stage 0: 4 (4.5%) Stage 1: 30 (33.7%) Stage 2: 7 (7.9%) Stage 3: 37 (41.6%) Stage 4: 11 (12.4%)		Stage 0: 1 (4.2%) Stage 1: 8 (33.3%) Stage 2: 1 (4.2%) Stage 3: 13 (54.2%) Stage 4: 1 (4.2%)	

Income	Up to $35,000: 27 (30.3%) $35,000–$65,000: 24 (27%) $65,001–$95,000: 10 (11.2%) $95,001–$125,000: 3 (3.4%) $125,001–$150,000: 4 (4.5%) >$150,000: 8 (9%) Prefer not to answer: 13 (14.6%)		Up to $35,000: 6 (25%) $35,000–$65,000: 6 (25%) $65,001–$95,000: 5 (20.8%) $95,001–$125,000: 1 (4.2%) $125,001–$150,000: 1 (4.2%) >$150,000: 1 (4.2%) Prefer not to answer: 4 (16.7%)	

Employment	Working full time: 8 (9%) Working part time: 8 (9%) Retired-pensioner: 36 (40.4%) Retired-self-funded retiree: 30 (33.7%) Unemployed currently: 4 (4.5%) Home duties: 3 (3.4%)		Working full time: 1 (4.2%) Working part time: 1 (4.2%) Retired-pensioner: 7 (29.2%) Retired-self-funded retiree: 14 (58.3%) Unemployed currently: 1 (4.2%) Home duties: 0 (0%)	

Physical activity days per week (*n* = 86)^#^	4.5 (2.2)	0–7	4.38 (2.2)	0–7

Effects of physical activity on pain (0 = completely relieves pain, 6 = large increase in pain)	2.9 (1.9)	0–6	3.33 (2.0)	0–6

Pain treatment use	Yes: 60 (67.4%) No: 29 (32.6%)		Yes: 16 (66.7%) No: 8 (33.3%)	

Average pain relief from pain treatment (% relief)^*∗*^	52 (21.5)	0–90	50 (20.0)	10–80

^#^Reason for missing data: participants entered days >7. ^*∗*^Data represent participants who responded yes to pain treatment use.

**Table 3:** Pain category and contributor data.

Measures	Domain	Whole sample *n* = 89		Interviewed sample *n* = 24	
		Mean (SD) and/or number (%)	Range	Mean (SD) and/or number (%)	Range
KPPQ total (/14)^*∗*^	Global pain measure	5.9 (2.7)	2–14	5.29 (2.2)	2–10

KPPQ D1; musculoskeletal (/1)^*∗*^	Global pain measure	0.9 (0.3)	0-1	1	1-1
		81 (91.0%)		24 (100%)	

KPPQ D2; chronic (/2)^*∗*^	Global pain measure	0.9 (0.7)	0–2	0.67 (0.6)	0–2
		59 (66.3%)		14 (58%)	

KPPQ D3; fluctuation related (/3)^*∗*^	Global pain measure	1.5 (1.0)	0–3	1.38 (1.0)	0–3
		72 (80.9%)		19 (79.2%)	

KPPQ D4; dyskinetic (/2)^*∗*^	Global pain measure	1.2 (0.7)	0–2	1.29 (0.8)	0–2
		77 (86.5%)		19 (79.2%)	

KPPQ D5; nocturnal (/3)^*∗*^	Global pain measure	0.3 (0.7)	0–3	0	0-0
		19 (21.3%)		0 (0%)	

KPPQ D6; orofacial (/2)^*∗*^	Global pain measure	0.6 (0.7)	0–2	0.5 (0.7)	0–2
		43 (48.3%)		10 (41.7%)	

KPPQ D7; discoloration and edema/swelling (/1)^*∗*^	Global pain measure	0.4 (0.5)	0-1	0.46 (0.5)	0-1
		36 (40.4%)		11 (45.8%)	

BPI pain severity score (/10)^*∗*^	Global pain measure	4.6 (1.7)	1.3–8.8	4.54 (1.7)	1.3–8.8

BPI pain interference score (/10)^*∗*^	Global pain measure	5.5 (2.2)	0.7–9.6	5.28 (1.6)	2.0–8.6

S-LANNS total (/24)^*∗*^	Peripheral neuropathy	8.6 (7.2)	0–24	9.33 (6.8)	0–24

CSI total (/100)^*∗*^	Central nociplastic change	47.5 (13.8)	15–91	46.7 (14.0)	15–71

PAS total (/48)^*∗*^	Emotional dysregulation or pathology	18.6 (9.3)	0–43	17.0 (9.2)	2–41

PCS total (/16)^*∗*^	Maladaptive cognitions	7.5 (3.6)	0–16	8.6 (3.4)	2–16

PFACTS total (/190)^*∗*^	Maladaptive cognitions	84.1 (53.8)	0–189	86.1 (47.7)	0–167

^*∗*^High score is worse (KPPQ: the King's PD Pain Questionnaire, BPI: Brief Pain Inventory, S-LANNS: Self-reported Leeds Assessment of Neuropathic Symptoms and Signs pain scale, CSI: Central Sensitization Inventory, PAS: Parkinson's Anxiety Scale, PCS: Brief Pain Catastrophizing Scale, and PFACTS: Pictorial Fear of Activity Scale).

**Table 4:** Pain treatment methods.

			Pain treatment use (all participants)				Pain treatment use (interview participants)
*Overall pain treatment methods*							
Users of one or more pain treatment types			60/89 (60%)				16/24 (66%)
Average pain relief from pain treatment			52%				50%

*Listed pain treatment types*							
Pain medication/anti-inflammatory			52/60 (86.7%)				15/16 (93.8%)
Parkinson's disease medications			13/60 (21.7%)				4/16 (25%)
Allied health professionals			13/60 (21.7%) physiotherapist, osteopath, chiropractor				3/16 (18.8%) (Physiotherapist and chiropractor)
Medical professionals/specialist			1/60 (2%)				0/16 (0%)
Massage/acupuncture			12/60 (20%)				3/16 (18.8%)
Heat			10/60 (16.7%)				3/16 (18.8%)
Physical activity (including exercise)			7/60 (11.7%)				2/16 (12.5%)
Anti-anxiety or anti-depressant medication			4/60 (6.7%)				1/16 (6.2%)
Other			13/60 (21.7%) (Cannabis, ice gel, cramp tablets, dementia and high blood pressure medication, cognitive coping, energy healing, Valium, rest, knee guard, spa)				3/16 (18.8%) (Cannabis, ice gel, cramp tablets, dementia, and high blood pressure medication)

*Pain treatment methods according to main pain contributor*							

Main pain contributor	Physical modalities	Cognitive strategies	HCP management	Medication use	Physical activity	Activity modification/pacing	Supporting quotes from participants with this main pain contributor

Maladaptive cognitions	✓	✓	NM	✓	✓	✓	*“I need to exercise to just keep on top of it” (P9, PD-3 yrs)* *“Pacing” (P10, PD-2 yrs)* *“Keeping positive” (P8, PD-9 yrs)* *“Meditation” (P16, PD-1 yr)* *“Massage” (P17, PD-14 yrs)* *varying forms of heat, i.e., “hot shower, hot packs” (P24, PD-6 yrs)* *“Botox” (P8, PD-9 yrs)* *“Steroids” (P13, PD-6 yrs)*
Central nociplastic	✓	✓	✓	✓	✓	✓	*“Just by massaging” (P24, PD-6 yrs)* *“I got an exercise physiologist and exercise has helped” (P11, PD-12 yrs)* *“Zone out from everything else and you go into almost a different part of your mind” (P10, PD-2 yrs)* *“With medication things are on the mend now” (P3, PD-2 yrs)* *“When I exercise I feel better” (P1, PD-17 yrs)* *“I'll sort of plan that sort of adapt what activities I am doing in the day” (P12, PD-1 yr)*
Peripheral neuropathic	✓	✓	✓	✓	✓	NM	*“I do massage in the chair” (P17, PD-14 yrs)* *“I use positive self-talk” (P1, PD-17 yrs)* *“I went to see a pain specialist” (P13, PD-6 yrs)* *“I need the medication.” (P7, PD-16 yrs)*
Emotional dysregulation or pathology	✓	NM	✓	✓	✓	NM	*“Lying down flat” (P9, PD-3 yrs)* *“Waiting for it [pain] to wear off” (P19, PD-13 yrs)* *“Stretching” (P19, PD-13 yrs)* *“tens machine” (P19, PD-13 yrs)* *“Cubes of ice” (P23, PD-3 yrs)* *“Specifically the physio on pain management” (P3, PD-2 yrs)* *Using varying forms of analgesics, i.e., “analgesic cream” (P19, PD-13 yrs), “Panadol” (P3, PD-2 yrs), “anti-inflammatories” (P9, PD-3 yrs)* *“Sleeping tablets” (P19, PD-13 yrs)*

HCP: healthcare professionals; NM: not mentioned.

## Data Availability

The survey and interview data used to support the findings of this study are included within the article and the supplementary information files. The raw data and full transcripts are not available publicly due to ethical reasons.

## Supplementary Materials

Supplementary Materials Supplementary material provided includes: Supplementary Figure: Study design, Survey, Supplementary Table 1: Descriptions of measurements (expanded version), Interview guide, and Supplementary Table 2: Supporting quotes.
