# Supplementary material for: A Mixed Methods Approach to Explore the Experience of Pain and Its Management in People with Parkinson's Disease
Source: Parkinsons Dis. 2024 May 25;2024:8515400. doi: 10.1155/2024/8515400 (PMC11144069; doi:10.1155/2024/8515400)
Supplement: Supplementary Materials — Supplementary material provided includes: Supplementary Figure: Study design, Survey, Supplementary Table 1: Descriptions of measurements (expanded version), Interview guide, and Supplementary Table 2: Supporting quotes. [file 8515400.f1.zip › a. PD_Supplementary Figure_Study design_v1.pdf]

Supplementary figure: Study design

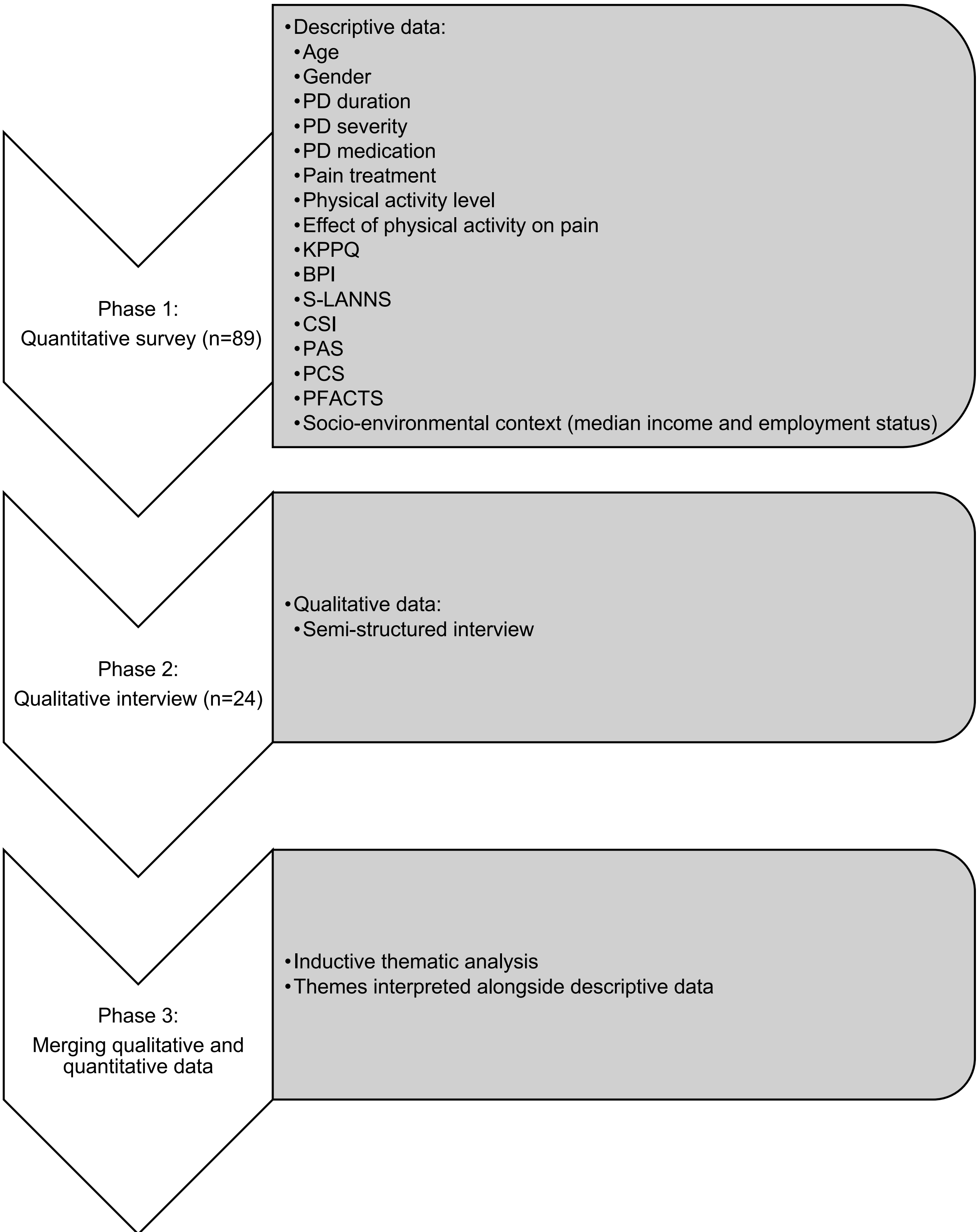

PD: Parkinson's disease, KPPQ: King's Parkinson's Disease Questionnaire, BPI: Brief Pain Inventory, S-LANNS: Self-reported Leeds Assessment of Neuropathic Symptoms and Signs pain scale, CSI: Central Sensitisation Inventory, PAS: Parkinson's Anxiety Scale, PCS: Pain Catastrophizing Scale, PFACTS: Pictorial Fear of Activity Scale
