# Supplementary material for: A Mixed Methods Approach to Explore the Experience of Pain and Its Management in People with Parkinson's Disease
Source: Parkinsons Dis. 2024 May 25;2024:8515400. doi: 10.1155/2024/8515400 (PMC11144069; doi:10.1155/2024/8515400)
Supplement: Supplementary Materials — Supplementary material provided includes: Supplementary Figure: Study design, Survey, Supplementary Table 1: Descriptions of measurements (expanded version), Interview guide, and Supplementary Table 2: Supporting quotes. [file 8515400.f1.zip › b. PD_Supplementary_Survey_V1_quality check.docx]

**Supplementary material – Survey**

**INSIGHTS INTO THE EXPERIENCE OF PAIN IN PARKINSON’S DISEASE: A MIXED METHODS STUDY**

Thank you for your interest in our study, please see the participant information statement for details *<hyperlinked>.*

| Do you consent to completing this survey? | |
| --- | --- |
|  | Yes, I have read the participant information statement. |
|  | No, I do not consent. Thank you for your time. The survey ends here. |

**Welcome to the Survey –** If you require assistance to complete the survey, please feel free to contact:

Vanessa Nguy at [vanessa.nguy@sydney.edu.au](mailto:vanessa.nguy@sydney.edu.au) or on XXXXX, or Dr. Natalie Allen on XXXXX

If you would prefer, you are allowed to have someone else assist you complete this survey but please ensure these responses are your own.

This survey gives you the option to save and continue later. If you leave your survey, simply click on the survey link (using the same internet browser and computer) to re-enter where you left off.

After selecting your responses to the questions on each page, ensure you scroll to the bottom (right side) of the page and click the forward arrow to continue.

**Inclusion Criteria**

Please tick (✔) your answer.

| Has a doctor diagnosed you with idiopathic Parkinson’s disease? | |
| --- | --- |
|  | Yes – proceed to the next question |
|  | No – Thank you for participating, the survey ends here |

| This question asks you to consider the severity of your pain on a scale from 0 to 10, where 0 is no pain and 10 is extreme pain. Have you experienced pain greater than 3/10 severity for ≥ 2 days/week in the last 3 months? | |
| --- | --- |
|  | Yes – proceed to the next question |
|  | No – Thank you for participating, the survey ends here |

| Have you ever had surgery on your spine (i.e. neck, back)? | |
| --- | --- |
|  | Yes – Thank you for participating, the survey ends here |
|  | No – proceed to the next question |

| Have you ever been diagnosed with a whiplash injury? | |
| --- | --- |
|  | Yes – Thank you for participating, the survey ends here |
|  | No – proceed to the next question |

| Have you ever been diagnosed with fibromyalgia? | |
| --- | --- |
|  | Yes – Thank you for participating, the survey ends here |
|  | No – proceed to the next question |

**Part 1. BPI**

On the diagrams below, please indicate the areas where you are currently feeling pain.

**FRONT LEFT SIDE BACK RIGHT SIDE**

**PALM OF LEFT HAND**

**PALM OF RIGHT HAND**

**BACK OF LEFT HAND**

**BACK OF RIGHT HAND**

**SOLE OF LEFT FOOT SOLE OF RIGHT FOOT**

**TOP OF LEFT FOOT TOP OF RIGHT FOOT**


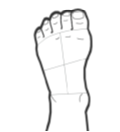


| Please rate your pain by ticking the one number that best describes you pain at its worst in the ***last week*** | | | | | | | | | | | | | | | |
| --- | --- | --- | --- | --- | --- | --- | --- | --- | --- | --- | --- | --- | --- | --- | --- |
| No pain | |  | |  | |  | |  | | Pain as bad as you can imagine | | | | | |
| 0 | 1 | | 2 | | 3 | | 4 | | 5 | | 6 | 7 | 8 | 9 | 10 |

| Please rate your pain by ticking the number that best describes your pain at its least in the ***last week*** | | | | | | | | | | | | | | | |
| --- | --- | --- | --- | --- | --- | --- | --- | --- | --- | --- | --- | --- | --- | --- | --- |
| No pain | |  | |  | |  | |  | | Pain as bad as you can imagine | | | | | |
| 0 | 1 | | 2 | | 3 | | 4 | | 5 | | 6 | 7 | 8 | 9 | 10 |

| Please rate your pain by ticking the one number that best describes your pain ***on average***. | | | | | | | | | | | | | | | |
| --- | --- | --- | --- | --- | --- | --- | --- | --- | --- | --- | --- | --- | --- | --- | --- |
| No pain | |  | |  | |  | |  | | Pain as bad as you can imagine | | | | | |
| 0 | 1 | | 2 | | 3 | | 4 | | 5 | | 6 | 7 | 8 | 9 | 10 |

| Please rate your pain by ticking the one number that tells how much pain you have ***right now***. | | | | | | | | | | | | | | | |
| --- | --- | --- | --- | --- | --- | --- | --- | --- | --- | --- | --- | --- | --- | --- | --- |
| No pain | |  | |  | |  | |  | | Pain as bad as you can imagine | | | | | |
| 0 | 1 | | 2 | | 3 | | 4 | | 5 | | 6 | 7 | 8 | 9 | 10 |

| Tick the one number that describes how, during the ***past week***, pain has interfered with your general activity | | | | | | | | | | | | | | | | |
| --- | --- | --- | --- | --- | --- | --- | --- | --- | --- | --- | --- | --- | --- | --- | --- | --- |
| Does not interfere | | | |  | |  |  | |  | |  | | Completely interferes | | | |
| 0 | 1 | 2 | 3 | | 4 | | | 5 | | 6 | | 7 | | 8 | 9 | 10 |

| Tick the one number that describes how, during the ***past week***, pain has interfered with your mood | | | | | | | | | | | | | | | | |
| --- | --- | --- | --- | --- | --- | --- | --- | --- | --- | --- | --- | --- | --- | --- | --- | --- |
| Does not interfere | | | |  | |  |  | |  | |  | | Completely interferes | | | |
| 0 | 1 | 2 | 3 | | 4 | | | 5 | | 6 | | 7 | | 8 | 9 | 10 |

| Tick the one number that describes how, during the ***past week***, pain has interfered with your walking ability | | | | | | | | | | | | | | | | |
| --- | --- | --- | --- | --- | --- | --- | --- | --- | --- | --- | --- | --- | --- | --- | --- | --- |
| Does not interfere | | | |  | |  |  | |  | |  | | Completely interferes | | | |
| 0 | 1 | 2 | 3 | | 4 | | | 5 | | 6 | | 7 | | 8 | 9 | 10 |

| Tick the one number that describes how, during the ***past week***, pain has interfered with your normal work (includes both outside the home and housework) | | | | | | | | | | | | | | | | |
| --- | --- | --- | --- | --- | --- | --- | --- | --- | --- | --- | --- | --- | --- | --- | --- | --- |
| Does not interfere | | | |  | |  |  | |  | |  | | Completely interferes | | | |
| 0 | 1 | 2 | 3 | | 4 | | | 5 | | 6 | | 7 | | 8 | 9 | 10 |

| Tick the one number that describes how, during the ***past week***, pain has interfered with your relations with other people | | | | | | | | | | | | | | | | |
| --- | --- | --- | --- | --- | --- | --- | --- | --- | --- | --- | --- | --- | --- | --- | --- | --- |
| Does not interfere | | | |  | |  |  | |  | |  | | Completely interferes | | | |
| 0 | 1 | 2 | 3 | | 4 | | | 5 | | 6 | | 7 | | 8 | 9 | 10 |

| Tick the one number that describes how, during the ***past week***, pain has interfered with your sleep | | | | | | | | | | | | | | | | |
| --- | --- | --- | --- | --- | --- | --- | --- | --- | --- | --- | --- | --- | --- | --- | --- | --- |
| Does not interfere | | | |  | |  |  | |  | |  | | Completely interferes | | | |
| 0 | 1 | 2 | 3 | | 4 | | | 5 | | 6 | | 7 | | 8 | 9 | 10 |

| Tick the one number that describes how, during the ***past week***, pain has interfered with your enjoyment of life | | | | | | | | | | | | | | | | |
| --- | --- | --- | --- | --- | --- | --- | --- | --- | --- | --- | --- | --- | --- | --- | --- | --- |
| Does not interfere | | | |  | |  |  | |  | |  | | Completely interferes | | | |
| 0 | 1 | 2 | 3 | | 4 | | | 5 | | 6 | | 7 | | 8 | 9 | 10 |

**Part 2. SLANSS**

| Think about the pain you have experienced over the ***past week***. Please tick (✔) the descriptions that best match your pain. These descriptions may, or may not, match your pain, no matter how severe it feels. | | |
| --- | --- | --- |
| In the area where you have pain, do you also have ‘pins and needles’, tingling or prickling sensations? | | |
|  | | NO I don’t get the sensations |
|  | | YES I do get these sensations |
| Does the painful area change colour (perhaps looks mottled or more red) when the pain is particularly bad? | | |
|  | | NO The pain does not affect the colour of my skin |
|  | | YES I have noticed that the pain does make my skin different from normal |
| Does your pain make the affected skin abnormally sensitive to touch? Getting unpleasant sensations or pain when lightly striking the skin might describe this | | |
|  | | NO The pain does not make my skin in that area abnormally sensitive to touch |
|  | | YES My skin in that area is particularly sensitive to touch |
| Does your pain come on suddenly and in bursts for no apparent reason when you are completely still? Words like ‘electric shocks’, jumping and bursting might describe this. | | |
|  | NO My pain doesn’t really feel like this | |
|  | YES I get these sensations often | |
| In the area where you have pain, does your skin feel unusually hot like a burning pain? | | |
|  | NO I don’t have burning pain | |
|  | YES I get these sensations often | |
| Gently rub the painful area with your index finger and then rub a non-painful area (for example the skin further away from the area, or on the opposite side). How does this rubbing feel in the painful area? | | |
|  | The pain area feels no different from the non-painful area | |
|  | I feel discomfort, like pins and needles, tingling or burning in the painful area that is different from the non-painful area | |
| Gently press on the painful area with your finger then gently press in the same way onto a non-painful area like you did in the last question. How does this feel in the painful area? | | |
|  | The pain area feels no different from the non-painful area | |
|  | I feel numbness or tenderness in the painful area that is different from the non-painful area | |

**Part 3. KPPQ**

| The movement symptoms of Parkinson’s are well known. However, other problems like pain can occur as part of the condition or its treatment. It is important to know about the specific type of your pain, particularly if it is troublesome for you.  Several types of pain are listed below:   - Please tick (✔) the box “Yes” if you have experienced this particular type of pain ***during the past month***. - If you have not experienced the type of pain in the ***past month*** tick the “No” box.   Please note that this survey only relates to the pain you experienced in the **last month**.  Have you experienced any of the following in the **last month**? | |
| --- | --- |
| Pain around the joints (including pain related to arthritis) | |
|  | NO |
|  | YES |
| Pain related to a specific internal organ (for example, pain around the liver, stomach or bowels) | |
|  | NO |
|  | YES |
| Generalised non-specific pain in your stomach area | |
|  | NO |
|  | YES |
| Non-specific pain deep within the body: a generalised constant, dull, aching pain | |
|  | NO |
|  | YES |
| Pain related to abnormal involuntary movements (dyskinetic pain) | |
|  | NO |
|  | YES |
| Painful muscle cramps in a specific region during “off” periods (when your medication is not working) | |
|  | NO |
|  | YES |
| Generalised pain during “off” periods (pain in the whole body or areas that are not affected by muscle cramps) | |
|  | NO |
|  | YES |
| Pain related to jerking leg movements during the night or an unpleasant burning sensation in the legs which improves with movement (restless legs syndrome) | |
|  | NO |
|  | YES |
| Pain related to difficulties when turning in bed at night | |
|  | NO |
|  | YES |
| Pain when chewing | |
|  | NO |
|  | YES |
| Pain related to grinding teeth during the night | |
|  | NO |
|  | YES |
| Burning sensation in your mouth | |
|  | NO |
|  | YES |
| Burning pain in the limbs (often associated with swelling or medication) | |
|  | NO |
|  | YES |
| Shooting pain/pins and needles down the limbs | |
|  | NO |
|  | YES |

**Part 4. PAS**

| **Please tick (✔)** **one box for each item below**  In the past ***four weeks***, to what extent did you experience the following symptoms? | |
| --- | --- |
| **A.1. Feeling anxious or nervous** | |
|  | Not at all, or never |
|  | Very mild, or rarely |
|  | Mild, or sometimes |
|  | Moderate, or often |
|  | Severe, or (nearly) always |
| **A.2. Feeling tense or stressed** | |
|  | Not at all, or never |
|  | Very mild, or rarely |
|  | Mild, or sometimes |
|  | Moderate, or often |
|  | Severe, or (nearly) always |
| **A.3. Being unable to relax** | |
|  | Not at all, or never |
|  | Very mild, or rarely |
|  | Mild, or sometimes |
|  | Moderate, or often |
|  | Severe, or (nearly) always |
| **A.4. Excessive worrying about everyday matters** | |
|  | Not at all, or never |
|  | Very mild, or rarely |
|  | Mild, or sometimes |
|  | Moderate, or often |
|  | Severe, or (nearly) always |
| **A.5. Fear of something bad, or even the worst, happening** | |
|  | Not at all, or never |
|  | Very mild, or rarely |
|  | Mild, or sometimes |
|  | Moderate, or often |
|  | Severe, or (nearly) always |

| **Please tick (✔)** **one box for each item below**  In the past ***four weeks***, did you experience episodes of the following symptoms? | |
| --- | --- |
| **B.1. Panic or intense fear** | |
|  | Never |
|  | Rarely |
|  | Sometimes |
|  | Often |
|  | Nearly always |
| **B.2. Shortness of breath** | |
|  | Never |
|  | Rarely |
|  | Sometimes |
|  | Often |
|  | Nearly always |
| **B.3. Heart palpitations or heart beating fast (not related to physical effort or activity)** | |
|  | Never |
|  | Rarely |
|  | Sometimes |
|  | Often |
|  | Nearly always |
| **B.4. Fear of losing control** | |
|  | Never |
|  | Rarely |
|  | Sometimes |
|  | Often |
|  | Nearly always |

| **Please tick (✔) one for each item below**  In the past ***four weeks***, to what extent did you fear or avoid the following situations? | |
| --- | --- |
| **C.1. Social situations (where one may be observed, or evaluated by others, such as speaking in public, or talking to unknown people)** | |
|  | Never |
|  | Rarely |
|  | Sometimes |
|  | Often |
|  | Nearly always |
| **C.2. Public settings (situations from which it may be difficult or embarrassing to escape, such as queues or lines, crowds, bridges, or public transportation)** | |
|  | Never |
|  | Rarely |
|  | Sometimes |
|  | Often |
|  | Nearly always |
| **C.3. Specific objects or situations (such as flying, heights, spiders or other animals, needles, or blood)** | |
|  | Never |
|  | Rarely |
|  | Sometimes |
|  | Often |
|  | Nearly always |

**Part 5. PCS**

| Everyone experiences painful situations at some point in their lives. Such experiences may include headaches, tooth pain, joint or muscle pain. People are often exposed to situations that may cause pain such as illness, injury, dental procedures or surgery.  We are interested in the types of thoughts and feelings that you have when you are in pain. Listed below are four statements describing different thoughts and feelings that may be associated with pain. Using the scale, please indicate by ticking (✔) one box for the degree to which you have these thoughts and feelings ***when you are experiencing pain***. | |
| --- | --- |
| It's awful and I feel that it overwhelms me | |
|  | Not at all |
|  | To a slight degree |
|  | To a moderate degree |
|  | To a great degree |
|  | All the time |
| I can’t seem to keep it out of my mind | |
|  | Not at all |
|  | To a slight degree |
|  | To a moderate degree |
|  | To a great degree |
|  | All the time |
| I keep thinking about how much it hurts | |
|  | Not at all |
|  | To a slight degree |
|  | To a moderate degree |
|  | To a great degree |
|  | All the time |
| I keep thinking about how badly I want the pain to stop | |
|  | Not at all |
|  | To a slight degree |
|  | To a moderate degree |
|  | To a great degree |
|  | All the time |

**Part 6. CSI**

| Please tick (✔) the one best response for each statement. | |
| --- | --- |
| I feel unrefreshed when I wake up in the morning | |
|  | Never |
|  | Rarely |
|  | Sometimes |
|  | Often |
|  | Always |
| My muscles feel stiff and achy | |
|  | Never |
|  | Rarely |
|  | Sometimes |
|  | Often |
|  | Always |
| I have anxiety attacks | |
|  | Never |
|  | Rarely |
|  | Sometimes |
|  | Often |
|  | Always |
| I grind or clench my teeth | |
|  | Never |
|  | Rarely |
|  | Sometimes |
|  | Often |
|  | Always |
| I have problems with diarrhea and/or constipation | |
|  | Never |
|  | Rarely |
|  | Sometimes |
|  | Often |
|  | Always |
| I need help in performing my daily activities | |
|  | Never |
|  | Rarely |
|  | Sometimes |
|  | Often |
|  | Always |
| I am sensitive to bright lights | |
|  | Never |
|  | Rarely |
|  | Sometimes |
|  | Often |
|  | Always |
| I get tired very easily when I am physically active | |
|  | Never |
|  | Rarely |
|  | Sometimes |
|  | Often |
|  | Always |
| I feel pain all over my body | |
|  | Never |
|  | Rarely |
|  | Sometimes |
|  | Often |
|  | Always |
| I have headaches | |
|  | Never |
|  | Rarely |
|  | Sometimes |
|  | Often |
|  | Always |
| I feel discomfort in my bladder and/or burning when I urinate | |
|  | Never |
|  | Rarely |
|  | Sometimes |
|  | Often |
|  | Always |
| I do not sleep well | |
|  | Never |
|  | Rarely |
|  | Sometimes |
|  | Often |
|  | Always |
| I have difficulty concentrating | |
|  | Never |
|  | Rarely |
|  | Sometimes |
|  | Often |
|  | Always |
| I have skin problems such as dryness, itchiness or rashes | |
|  | Never |
|  | Rarely |
|  | Sometimes |
|  | Often |
|  | Always |
| Stress makes my physical symptoms get worse | |
|  | Never |
|  | Rarely |
|  | Sometimes |
|  | Often |
|  | Always |
| I feel sad or depressed | |
|  | Never |
|  | Rarely |
|  | Sometimes |
|  | Often |
|  | Always |
| I have low energy | |
|  | Never |
|  | Rarely |
|  | Sometimes |
|  | Often |
|  | Always |
| I have muscle tension in my neck and shoulders | |
|  | Never |
|  | Rarely |
|  | Sometimes |
|  | Often |
|  | Always |
| I have pain in my jaw | |
|  | Never |
|  | Rarely |
|  | Sometimes |
|  | Often |
|  | Always |
| Certain smells, such as perfumes, make me feel dizzy and nauseated | |
|  | Never |
|  | Rarely |
|  | Sometimes |
|  | Often |
|  | Always |
| I have to urinate frequently | |
|  | Never |
|  | Rarely |
|  | Sometimes |
|  | Often |
|  | Always |
| My legs feel uncomfortable and restless when I am trying to go to sleep at night | |
|  | Never |
|  | Rarely |
|  | Sometimes |
|  | Often |
|  | Always |
| I have difficulty remembering things | |
|  | Never |
|  | Rarely |
|  | Sometimes |
|  | Often |
|  | Always |
| I suffered trauma as a child | |
|  | Never |
|  | Rarely |
|  | Sometimes |
|  | Often |
|  | Always |
| I have pain in my pelvic area | |
|  | Never |
|  | Rarely |
|  | Sometimes |
|  | Often |
|  | Always |

**Part 7. PFACTS**

We will now show you 19 pictures of a person making different movements. For each picture, please tick one number that describes how worried or fearful of pain or injury you would be if you were to carry out the activity shown in the picture.

| 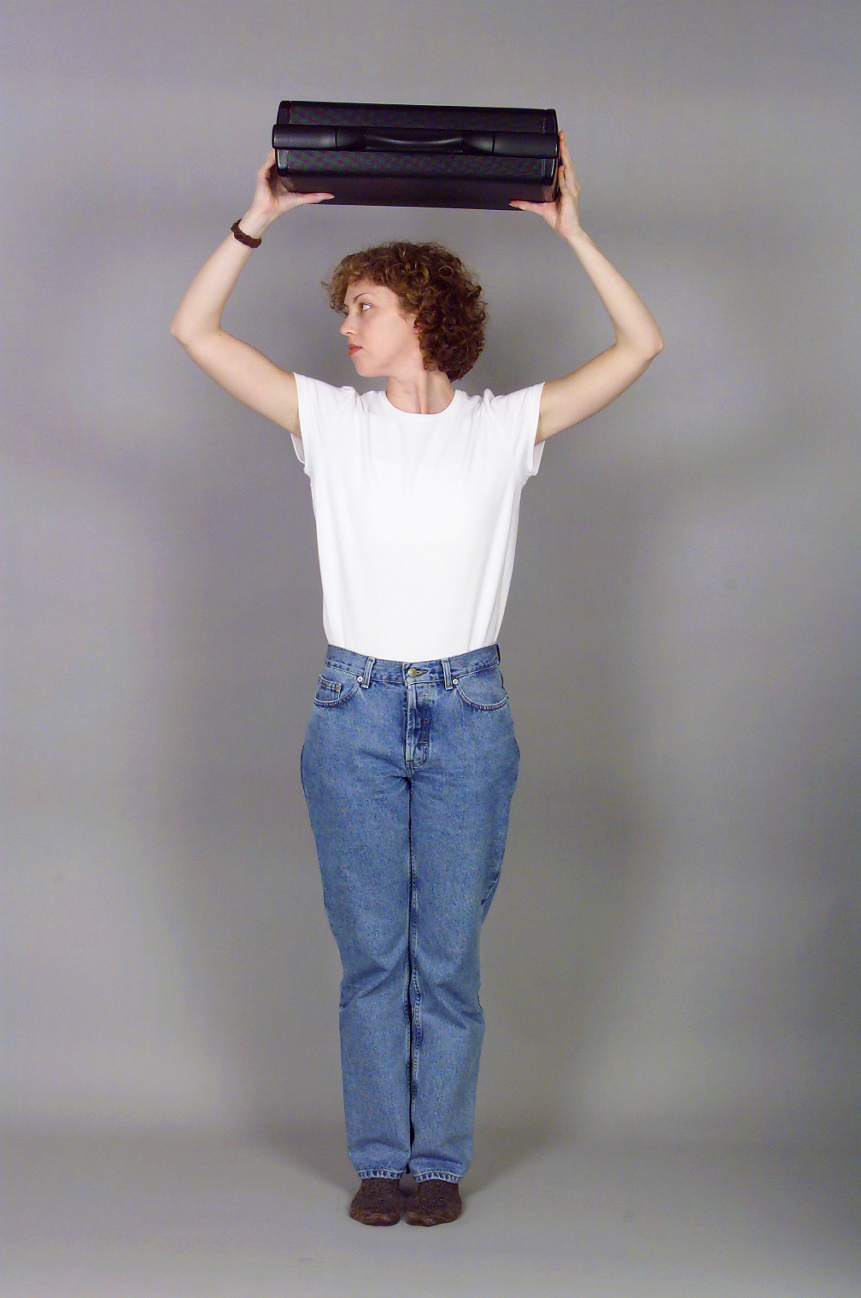  Tick one number that describes how worried or fearful of pain or injury you would be to carry out the activity shown in the picture above. | | | | | | | | | | | | | | | | |
| --- | --- | --- | --- | --- | --- | --- | --- | --- | --- | --- | --- | --- | --- | --- | --- | --- |
| No fear at all | | | |  | |  |  | |  | |  | | Extremely fearful | | | |
| 0 | 1 | 2 | 3 | | 4 | | | 5 | | 6 | | 7 | | 8 | 9 | 10 |

| 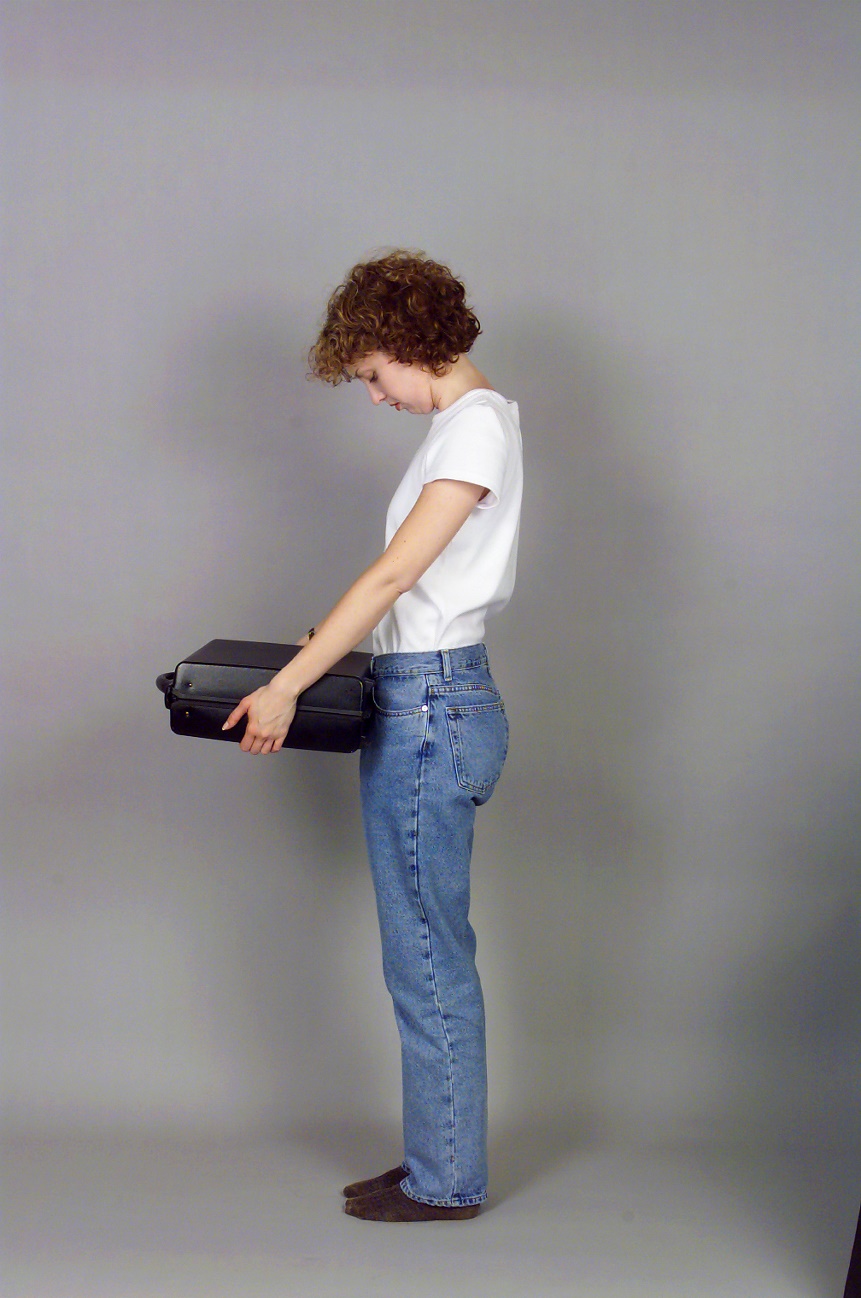  Tick one number that describes how worried or fearful of pain or injury you would be to carry out the activity shown in the picture above. | | | | | | | | | | | | | | | | |
| --- | --- | --- | --- | --- | --- | --- | --- | --- | --- | --- | --- | --- | --- | --- | --- | --- |
| No fear at all | | | |  | |  |  | |  | |  | | Extremely fearful | | | |
| 0 | 1 | 2 | 3 | | 4 | | | 5 | | 6 | | 7 | | 8 | 9 | 10 |

| 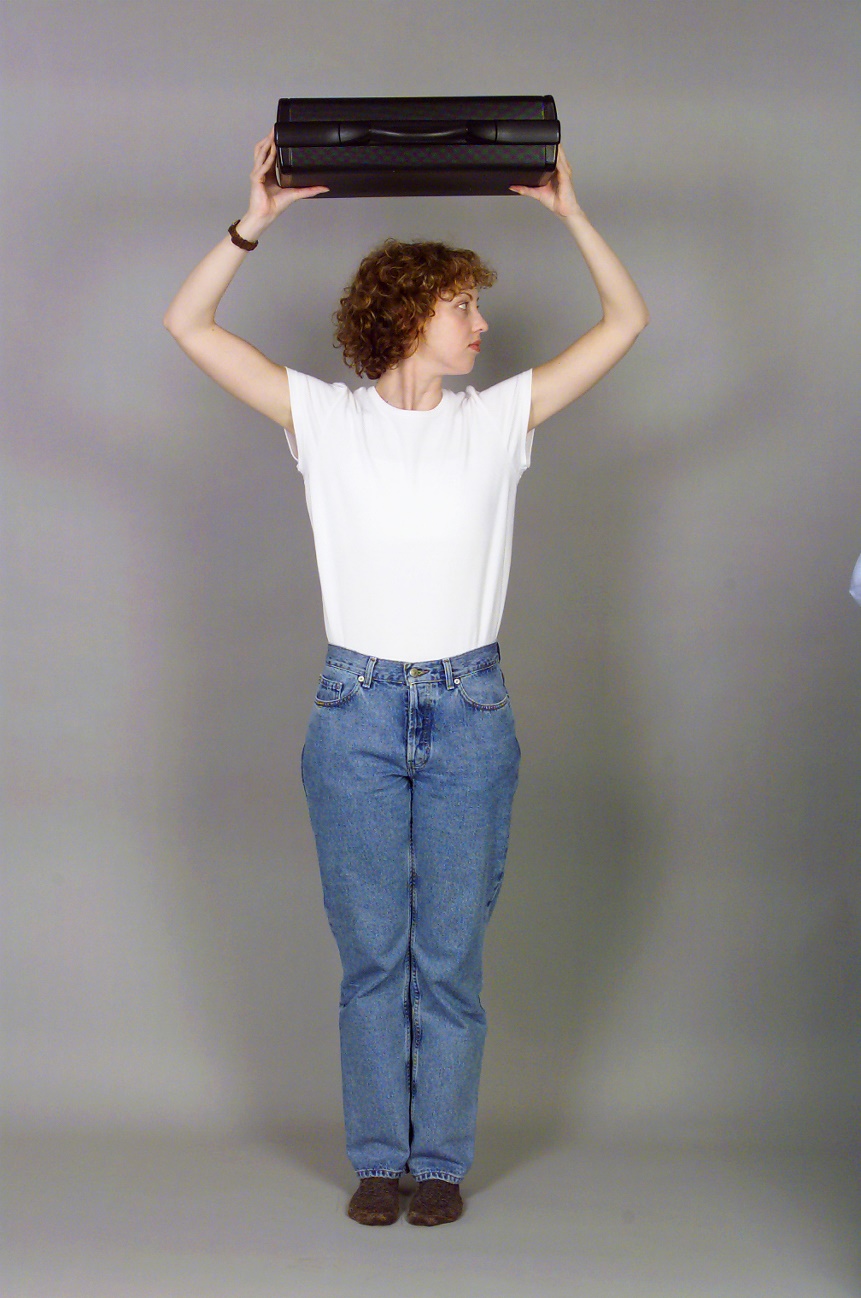  Tick one number that describes how worried or fearful of pain or injury you would be to carry out the activity shown in the picture above. | | | | | | | | | | | | | | | | |
| --- | --- | --- | --- | --- | --- | --- | --- | --- | --- | --- | --- | --- | --- | --- | --- | --- |
| No fear at all | | | |  | |  |  | |  | |  | | Extremely fearful | | | |
| 0 | 1 | 2 | 3 | | 4 | | | 5 | | 6 | | 7 | | 8 | 9 | 10 |

| 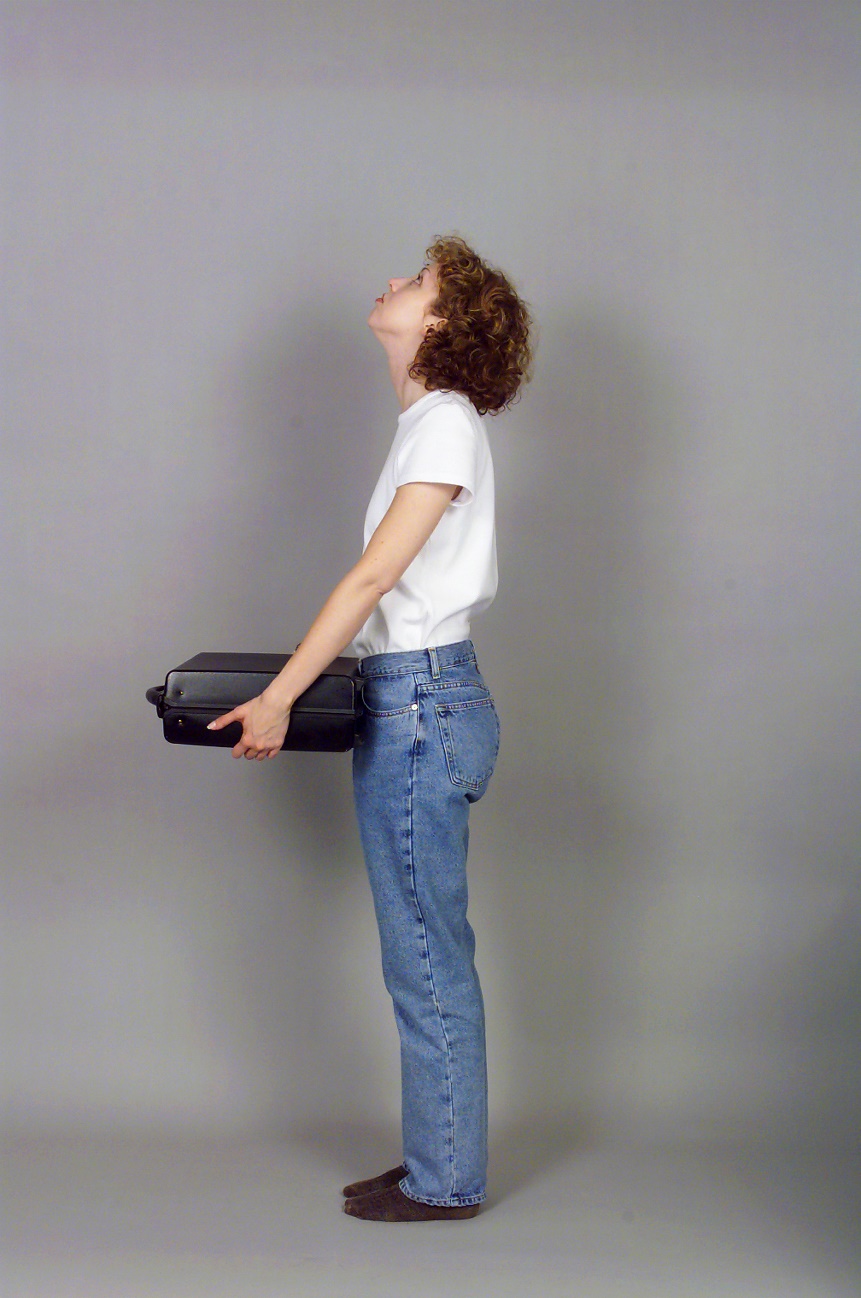  Tick one number that describes how worried or fearful of pain or injury you would be to carry out the activity shown in the picture above. | | | | | | | | | | | | | | | | |
| --- | --- | --- | --- | --- | --- | --- | --- | --- | --- | --- | --- | --- | --- | --- | --- | --- |
| No fear at all | | | |  | |  |  | |  | |  | | Extremely fearful | | | |
| 0 | 1 | 2 | 3 | | 4 | | | 5 | | 6 | | 7 | | 8 | 9 | 10 |

| 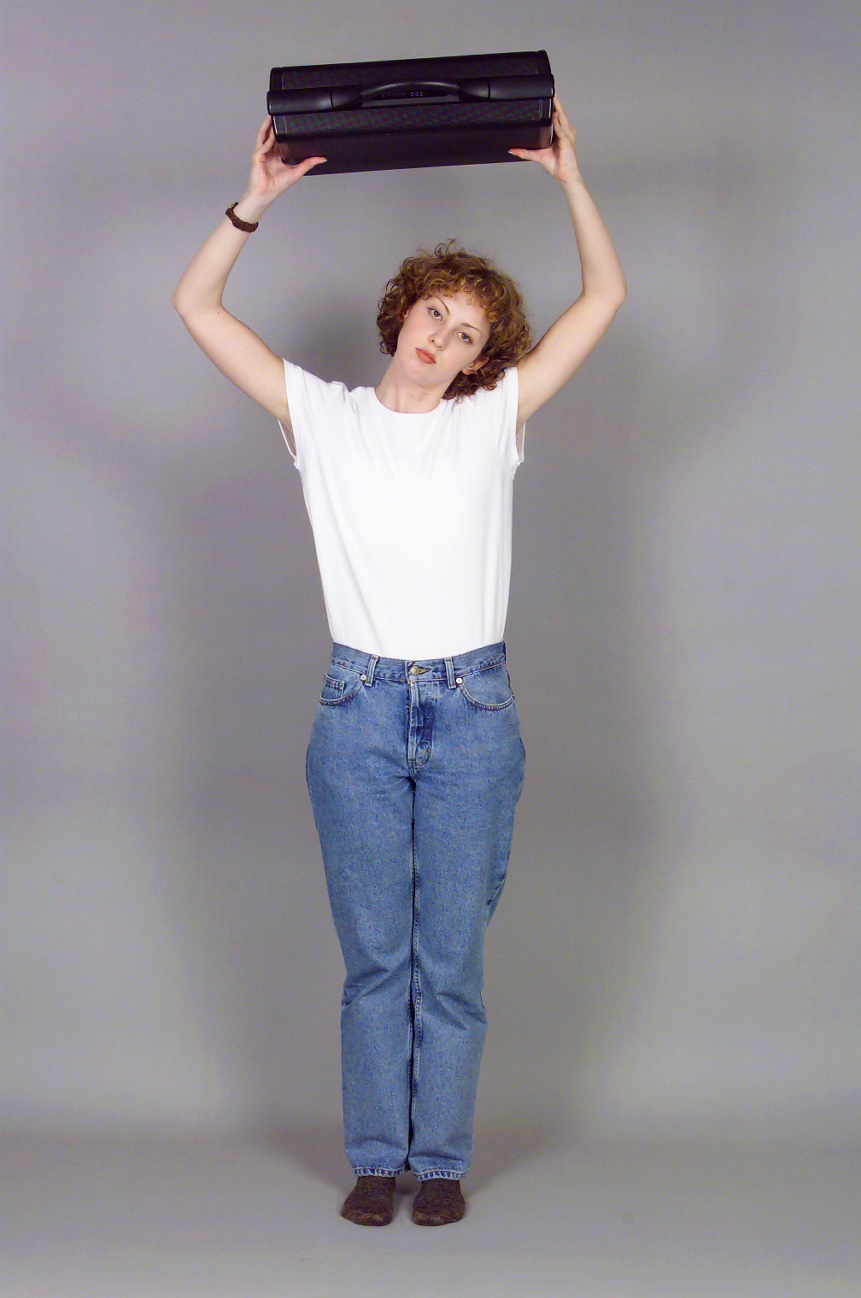  Tick one number that describes how worried or fearful of pain or injury you would be to carry out the activity shown in the picture above. | | | | | | | | | | | | | | | | |
| --- | --- | --- | --- | --- | --- | --- | --- | --- | --- | --- | --- | --- | --- | --- | --- | --- |
| No fear at all | | | |  | |  |  | |  | |  | | Extremely fearful | | | |
| 0 | 1 | 2 | 3 | | 4 | | | 5 | | 6 | | 7 | | 8 | 9 | 10 |

| 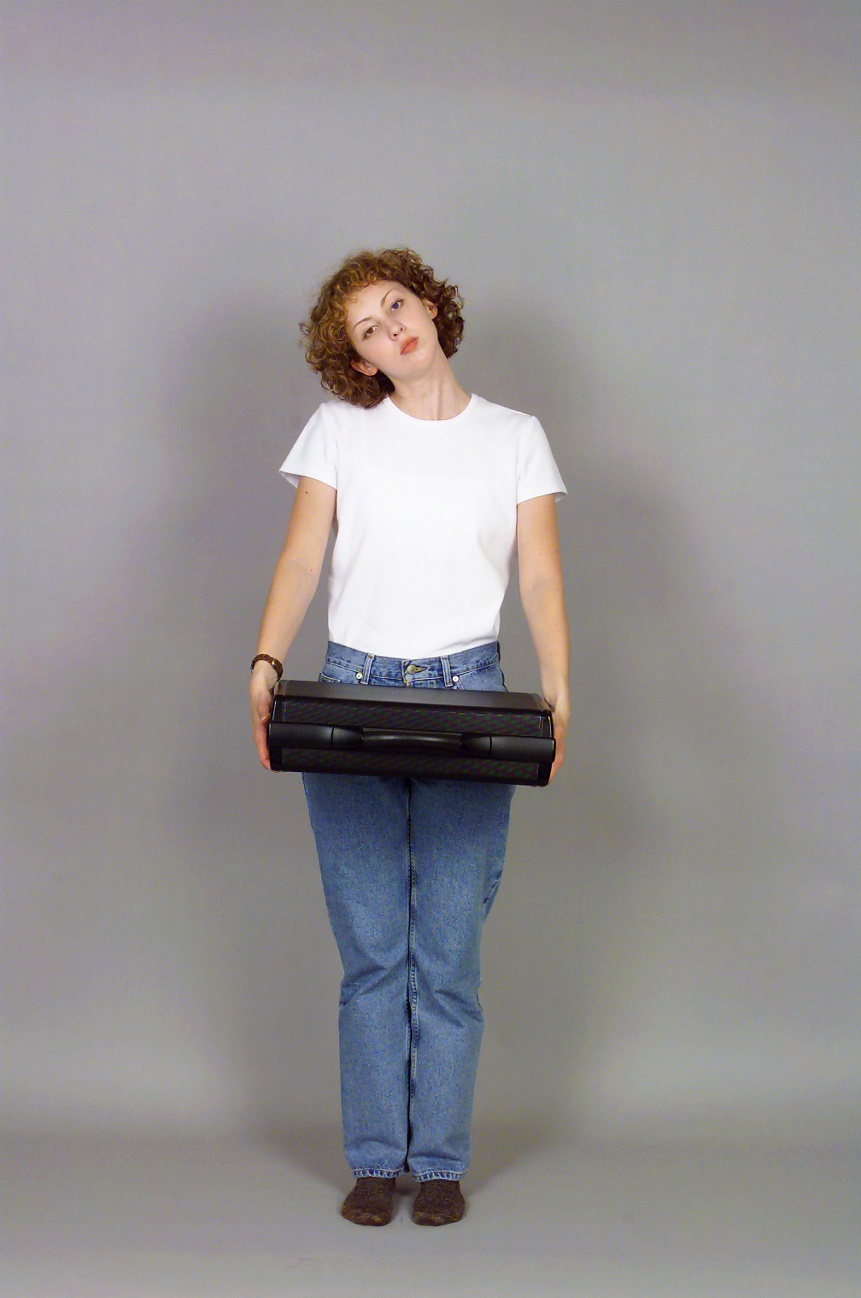  Tick one number that describes how worried or fearful of pain or injury you would be to carry out the activity shown in the picture above. | | | | | | | | | | | | | | | | |
| --- | --- | --- | --- | --- | --- | --- | --- | --- | --- | --- | --- | --- | --- | --- | --- | --- |
| No fear at all | | | |  | |  |  | |  | |  | | Extremely fearful | | | |
| 0 | 1 | 2 | 3 | | 4 | | | 5 | | 6 | | 7 | | 8 | 9 | 10 |

| 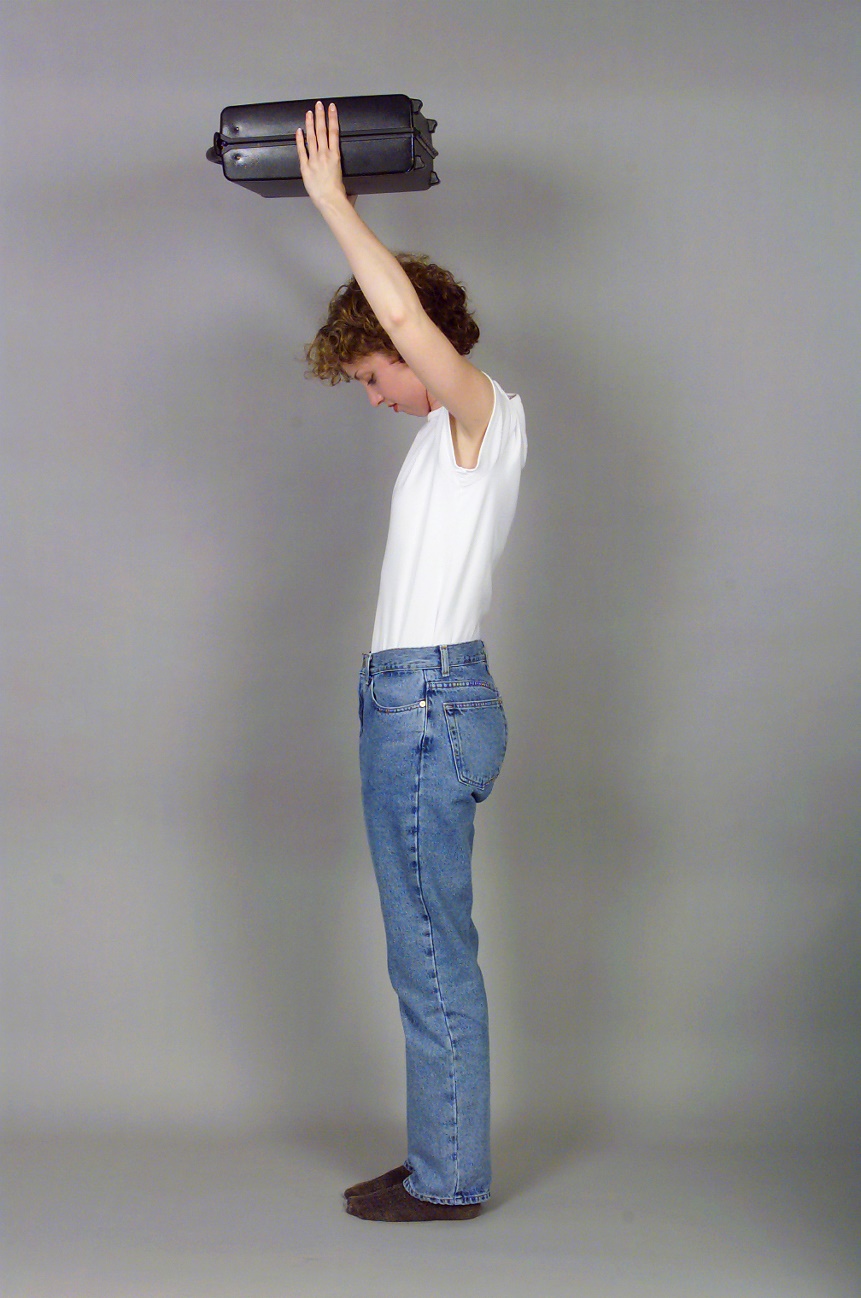  Tick one number that describes how worried or fearful of pain or injury you would be to carry out the activity shown in the picture above. | | | | | | | | | | | | | | | | |
| --- | --- | --- | --- | --- | --- | --- | --- | --- | --- | --- | --- | --- | --- | --- | --- | --- |
| No fear at all | | | |  | |  |  | |  | |  | | Extremely fearful | | | |
| 0 | 1 | 2 | 3 | | 4 | | | 5 | | 6 | | 7 | | 8 | 9 | 10 |

| 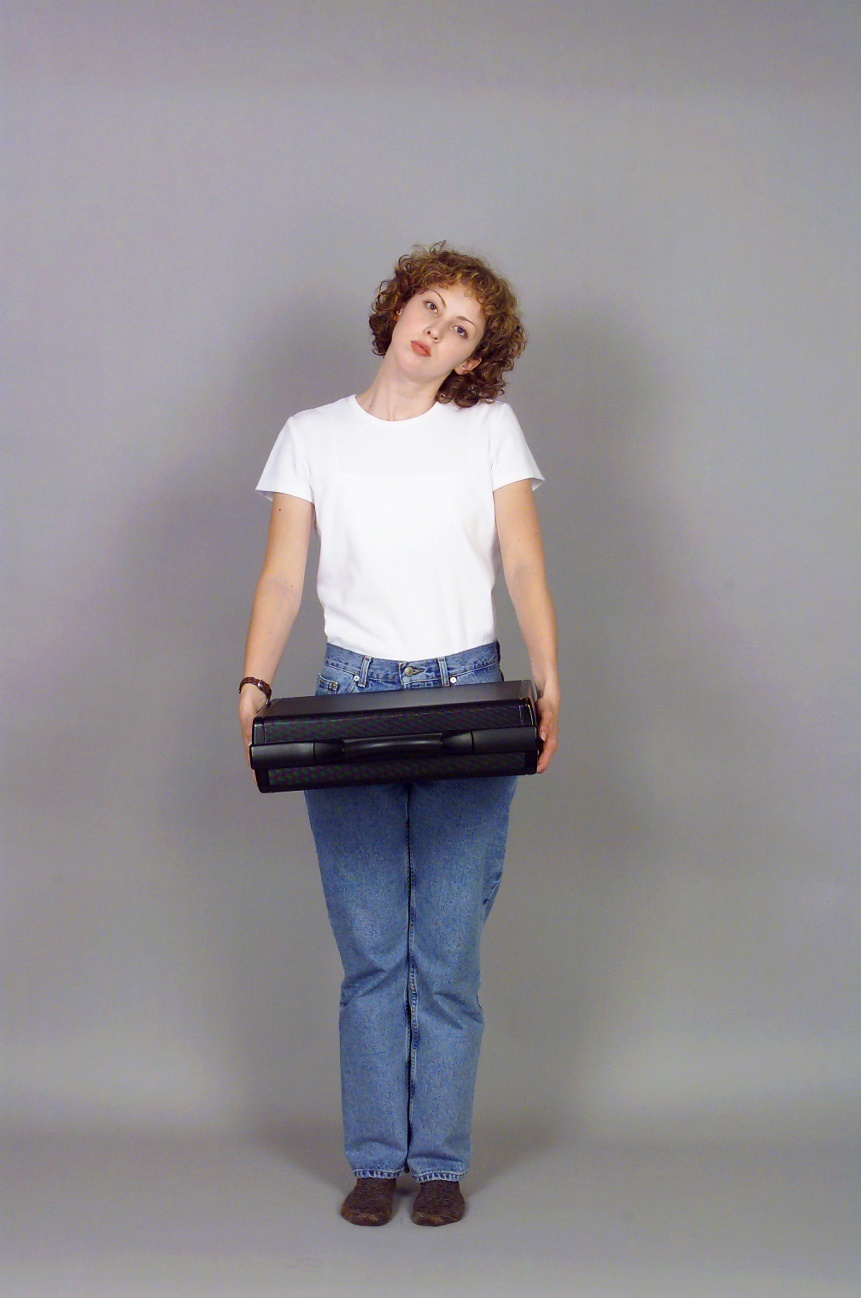  Tick one number that describes how worried or fearful of pain or injury you would be to carry out the activity shown in the picture above. | | | | | | | | | | | | | | | | |
| --- | --- | --- | --- | --- | --- | --- | --- | --- | --- | --- | --- | --- | --- | --- | --- | --- |
| No fear at all | | | |  | |  |  | |  | |  | | Extremely fearful | | | |
| 0 | 1 | 2 | 3 | | 4 | | | 5 | | 6 | | 7 | | 8 | 9 | 10 |

| 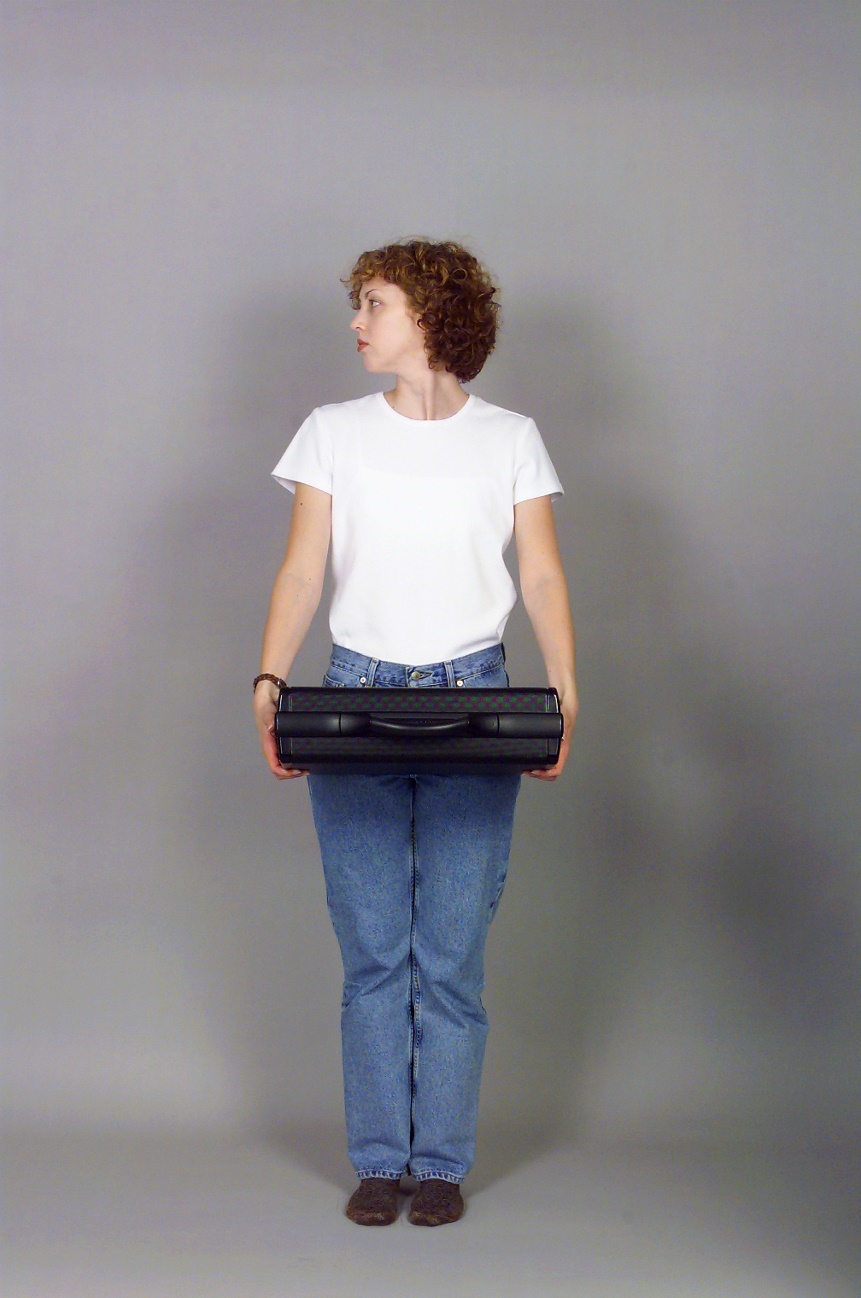  Tick one number that describes how worried or fearful of pain or injury you would be to carry out the activity shown in the picture above. | | | | | | | | | | | | | | | | |
| --- | --- | --- | --- | --- | --- | --- | --- | --- | --- | --- | --- | --- | --- | --- | --- | --- |
| No fear at all | | | |  | |  |  | |  | |  | | Extremely fearful | | | |
| 0 | 1 | 2 | 3 | | 4 | | | 5 | | 6 | | 7 | | 8 | 9 | 10 |

| 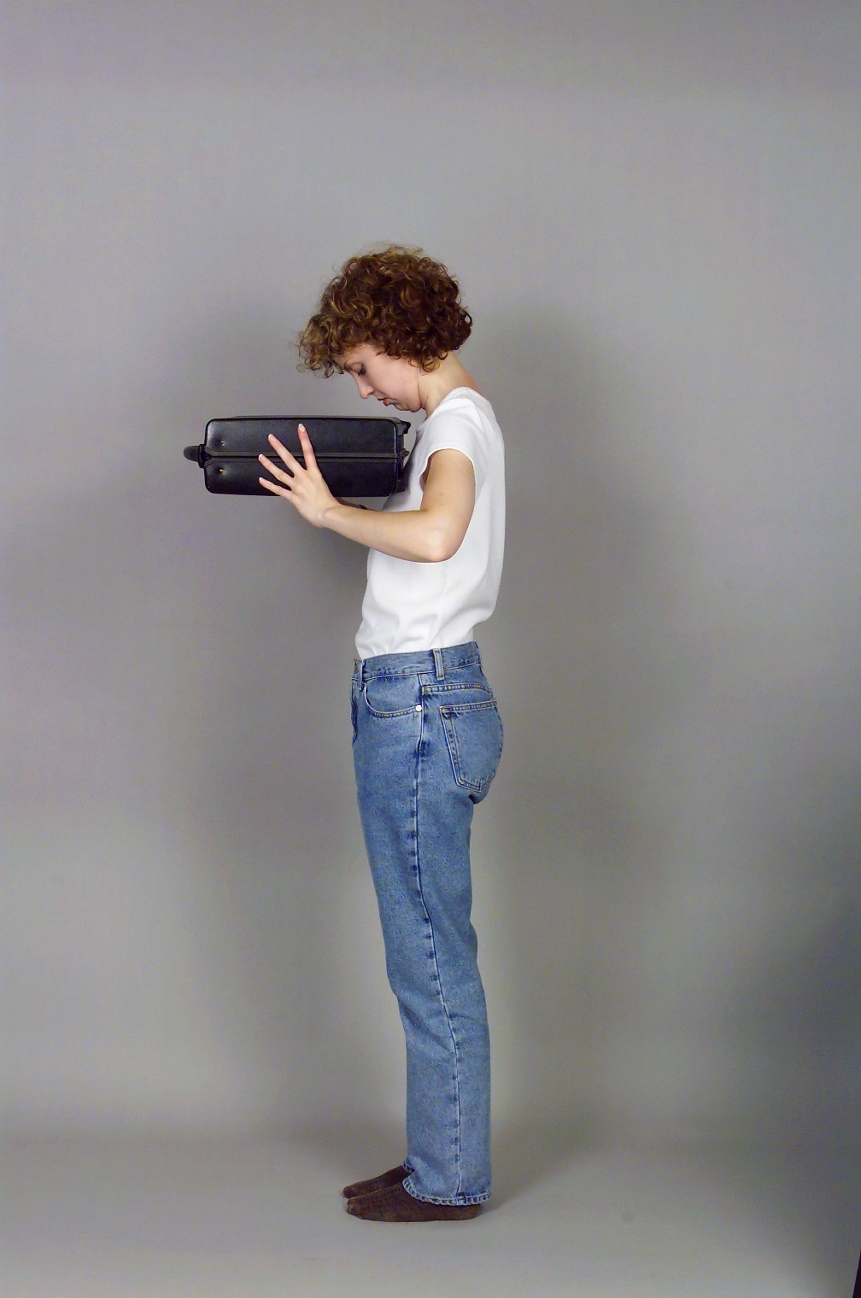  Tick one number that describes how worried or fearful of pain or injury you would be to carry out the activity shown in the picture above. | | | | | | | | | | | | | | | | |
| --- | --- | --- | --- | --- | --- | --- | --- | --- | --- | --- | --- | --- | --- | --- | --- | --- |
| No fear at all | | | |  | |  |  | |  | |  | | Extremely fearful | | | |
| 0 | 1 | 2 | 3 | | 4 | | | 5 | | 6 | | 7 | | 8 | 9 | 10 |

| 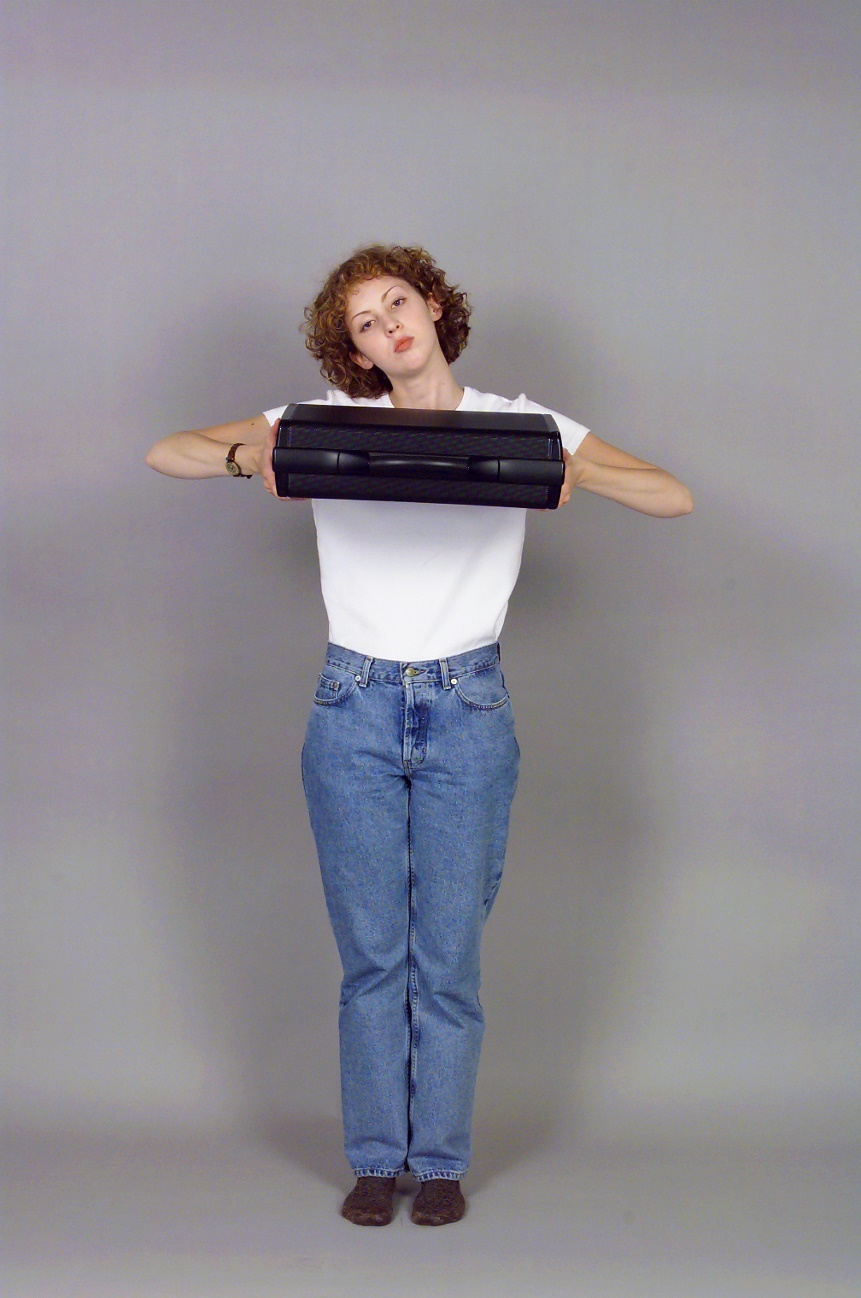  Tick one number that describes how worried or fearful of pain or injury you would be to carry out the activity shown in the picture above. | | | | | | | | | | | | | | | | |
| --- | --- | --- | --- | --- | --- | --- | --- | --- | --- | --- | --- | --- | --- | --- | --- | --- |
| No fear at all | | | |  | |  |  | |  | |  | | Extremely fearful | | | |
| 0 | 1 | 2 | 3 | | 4 | | | 5 | | 6 | | 7 | | 8 | 9 | 10 |

| 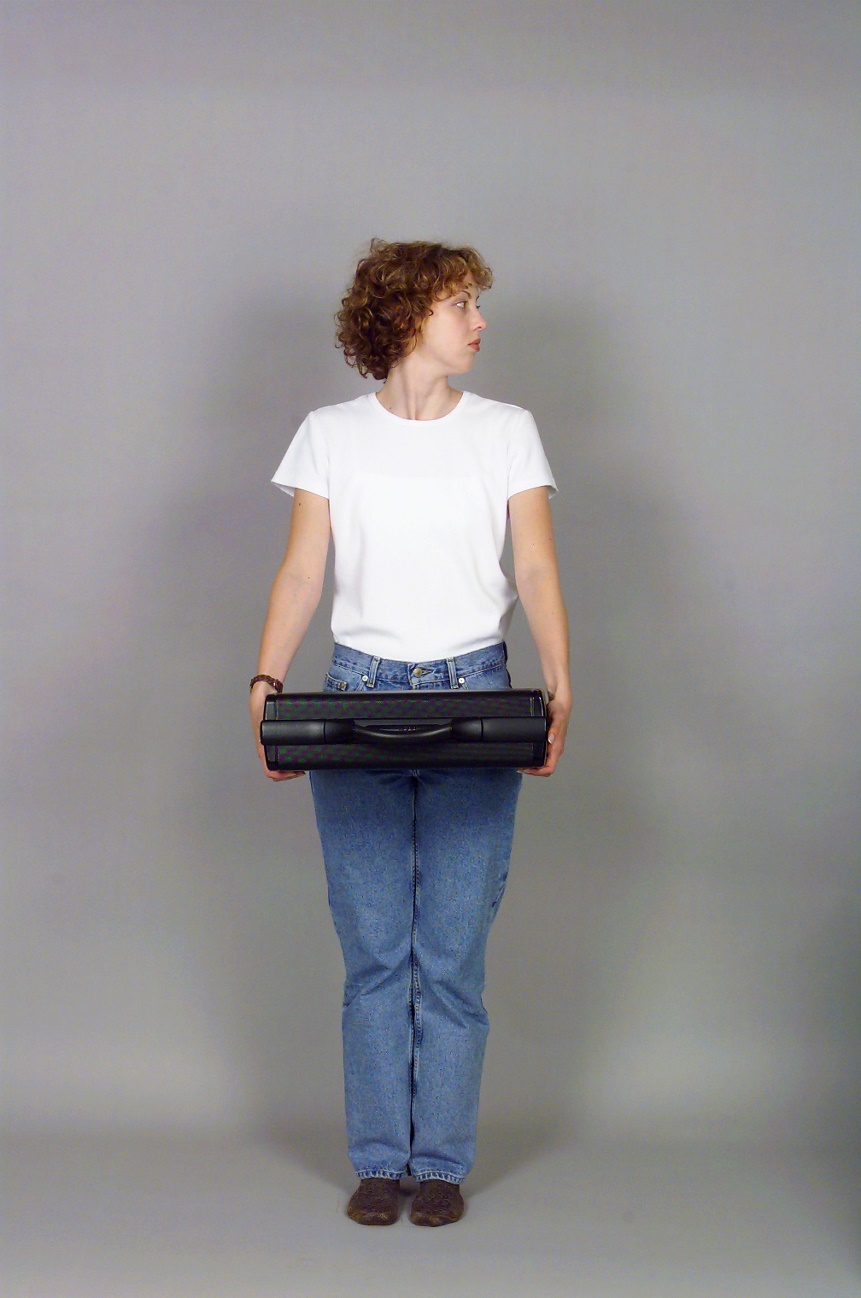  Tick one number that describes how worried or fearful of pain or injury you would be to carry out the activity shown in the picture above. | | | | | | | | | | | | | | | | |
| --- | --- | --- | --- | --- | --- | --- | --- | --- | --- | --- | --- | --- | --- | --- | --- | --- |
| No fear at all | | | |  | |  |  | |  | |  | | Extremely fearful | | | |
| 0 | 1 | 2 | 3 | | 4 | | | 5 | | 6 | | 7 | | 8 | 9 | 10 |

| 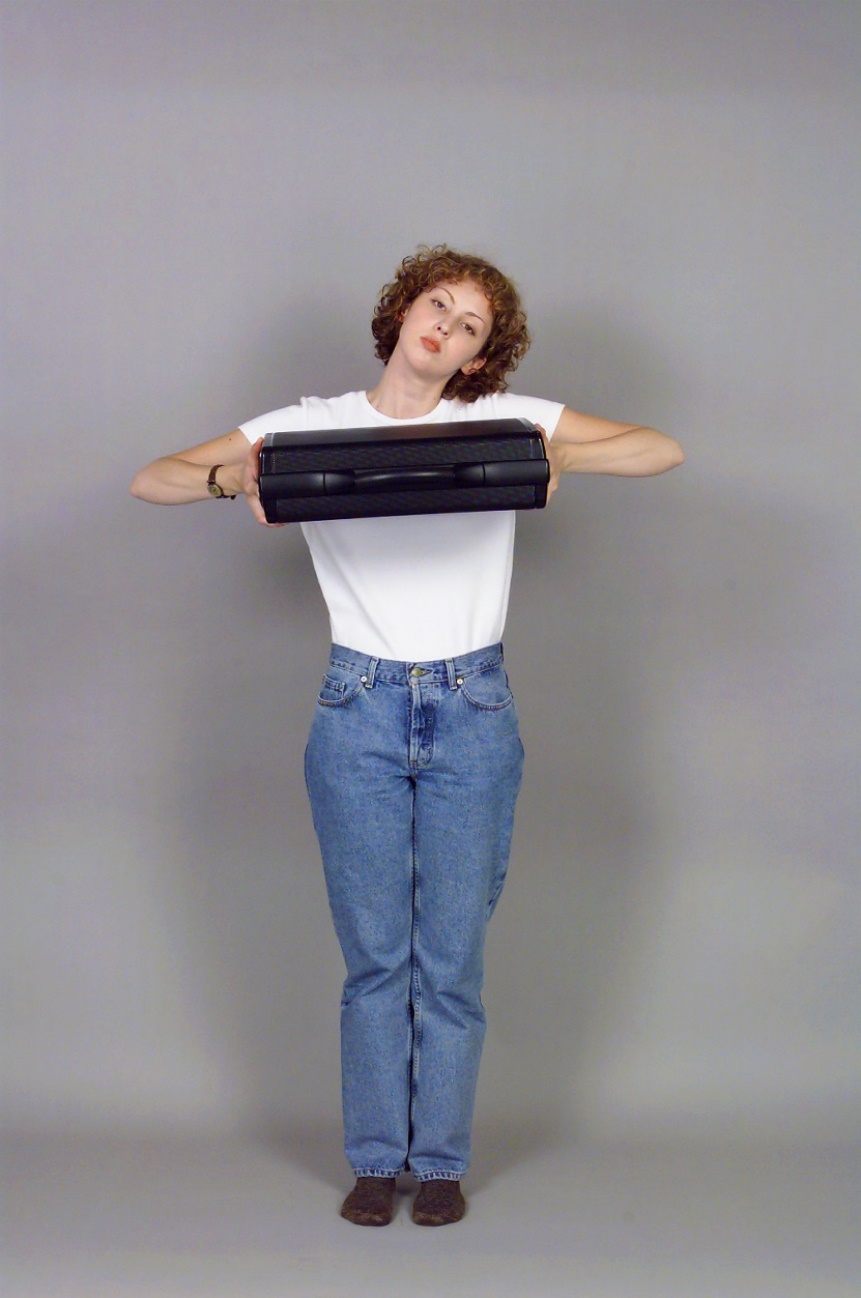  Tick one number that describes how worried or fearful of pain or injury you would be to carry out the activity shown in the picture above. | | | | | | | | | | | | | | | | |
| --- | --- | --- | --- | --- | --- | --- | --- | --- | --- | --- | --- | --- | --- | --- | --- | --- |
| No fear at all | | | |  | |  |  | |  | |  | | Extremely fearful | | | |
| 0 | 1 | 2 | 3 | | 4 | | | 5 | | 6 | | 7 | | 8 | 9 | 10 |

| 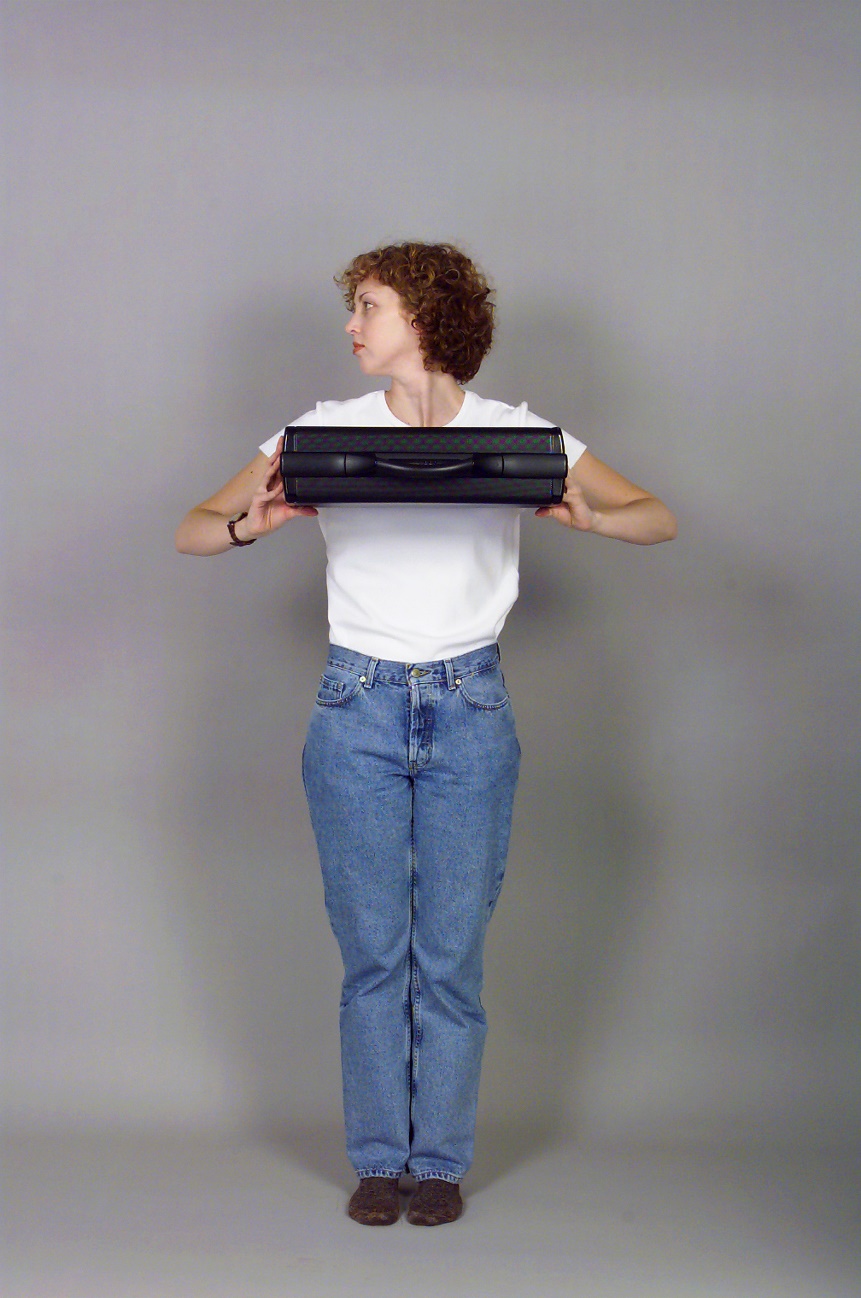  Tick one number that describes how worried or fearful of pain or injury you would be to carry out the activity shown in the picture above. | | | | | | | | | | | | | | | | |
| --- | --- | --- | --- | --- | --- | --- | --- | --- | --- | --- | --- | --- | --- | --- | --- | --- |
| No fear at all | | | |  | |  |  | |  | |  | | Extremely fearful | | | |
| 0 | 1 | 2 | 3 | | 4 | | | 5 | | 6 | | 7 | | 8 | 9 | 10 |

| 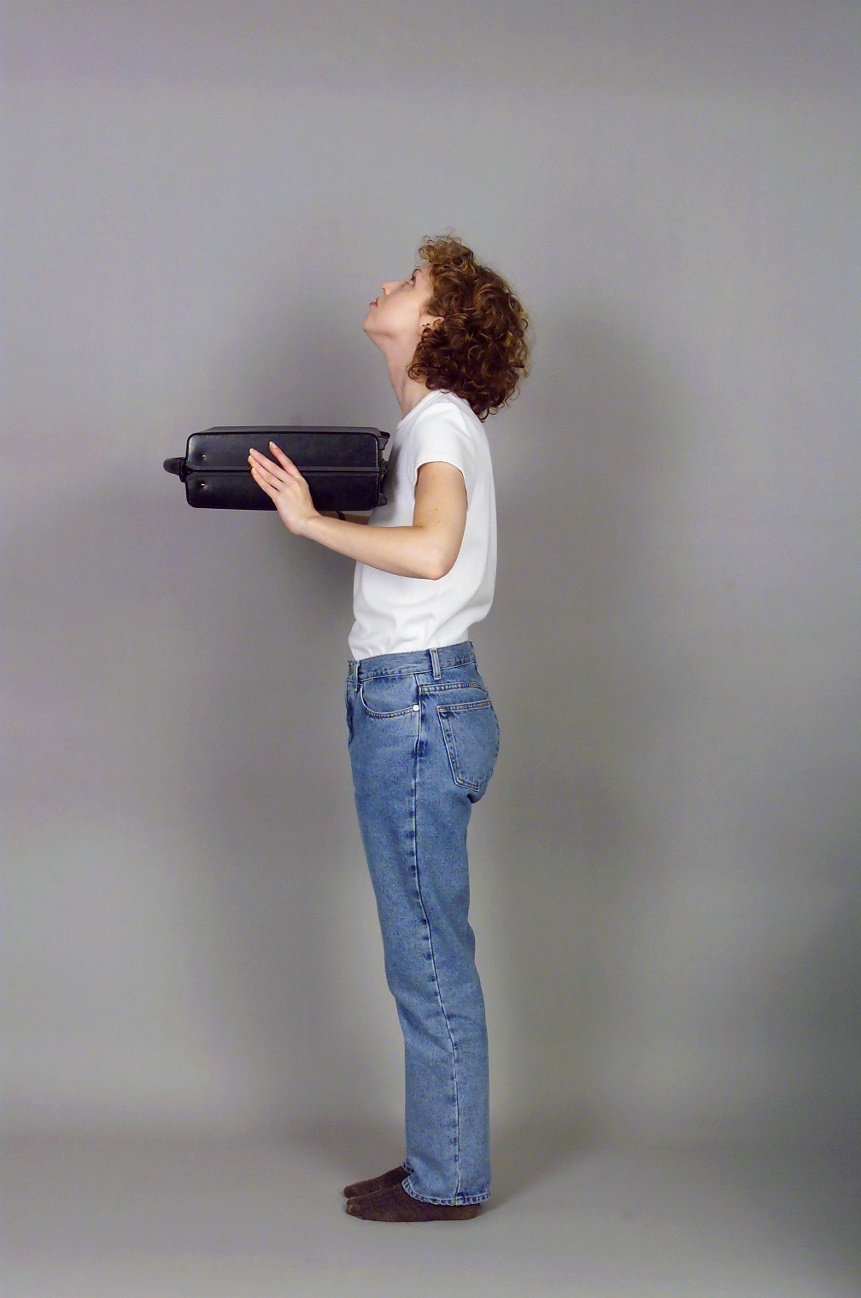  Tick one number that describes how worried or fearful of pain or injury you would be to carry out the activity shown in the picture above. | | | | | | | | | | | | | | | | |
| --- | --- | --- | --- | --- | --- | --- | --- | --- | --- | --- | --- | --- | --- | --- | --- | --- |
| No fear at all | | | |  | |  |  | |  | |  | | Extremely fearful | | | |
| 0 | 1 | 2 | 3 | | 4 | | | 5 | | 6 | | 7 | | 8 | 9 | 10 |

| 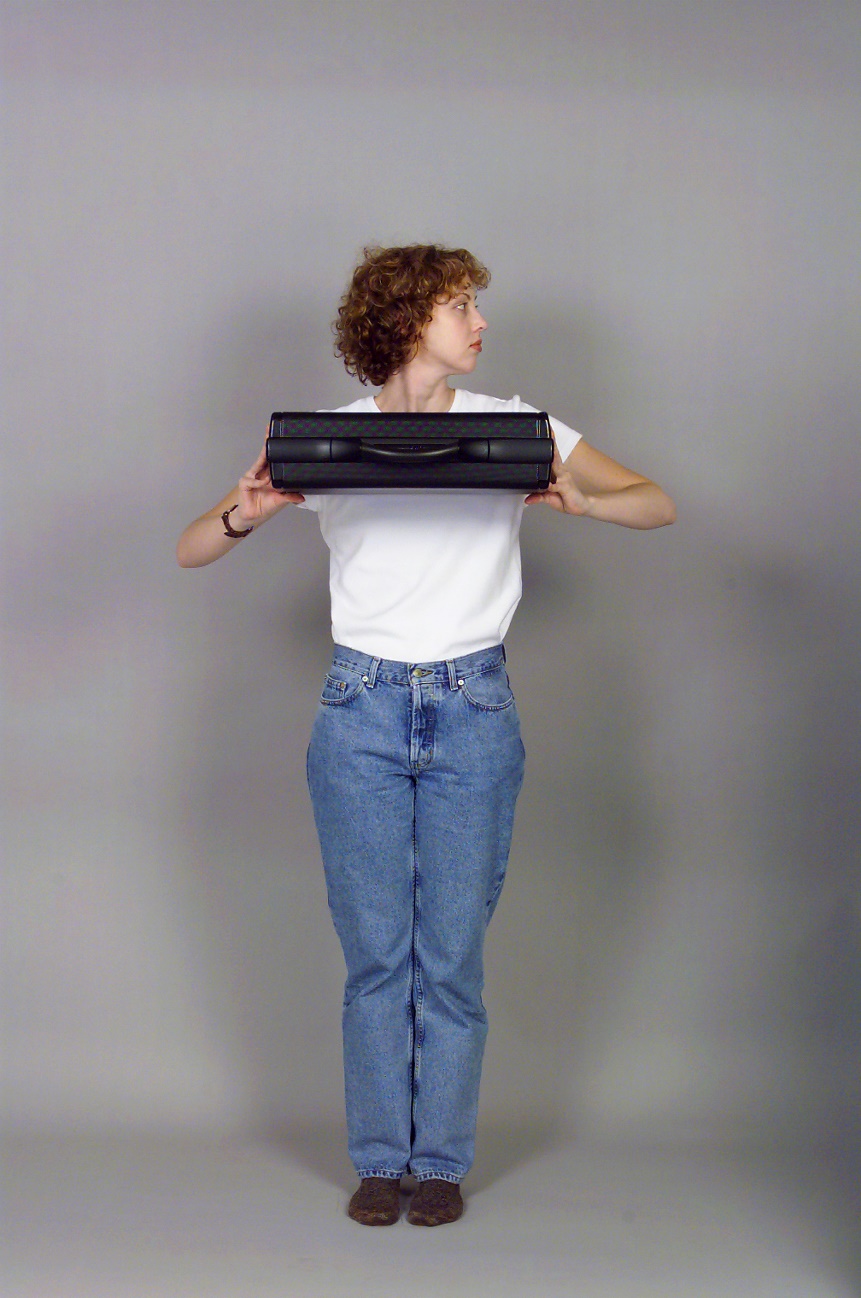  Tick one number that describes how worried or fearful of pain or injury you would be to carry out the activity shown in the picture above. | | | | | | | | | | | | | | | | |
| --- | --- | --- | --- | --- | --- | --- | --- | --- | --- | --- | --- | --- | --- | --- | --- | --- |
| No fear at all | | | |  | |  |  | |  | |  | | Extremely fearful | | | |
| 0 | 1 | 2 | 3 | | 4 | | | 5 | | 6 | | 7 | | 8 | 9 | 10 |

| 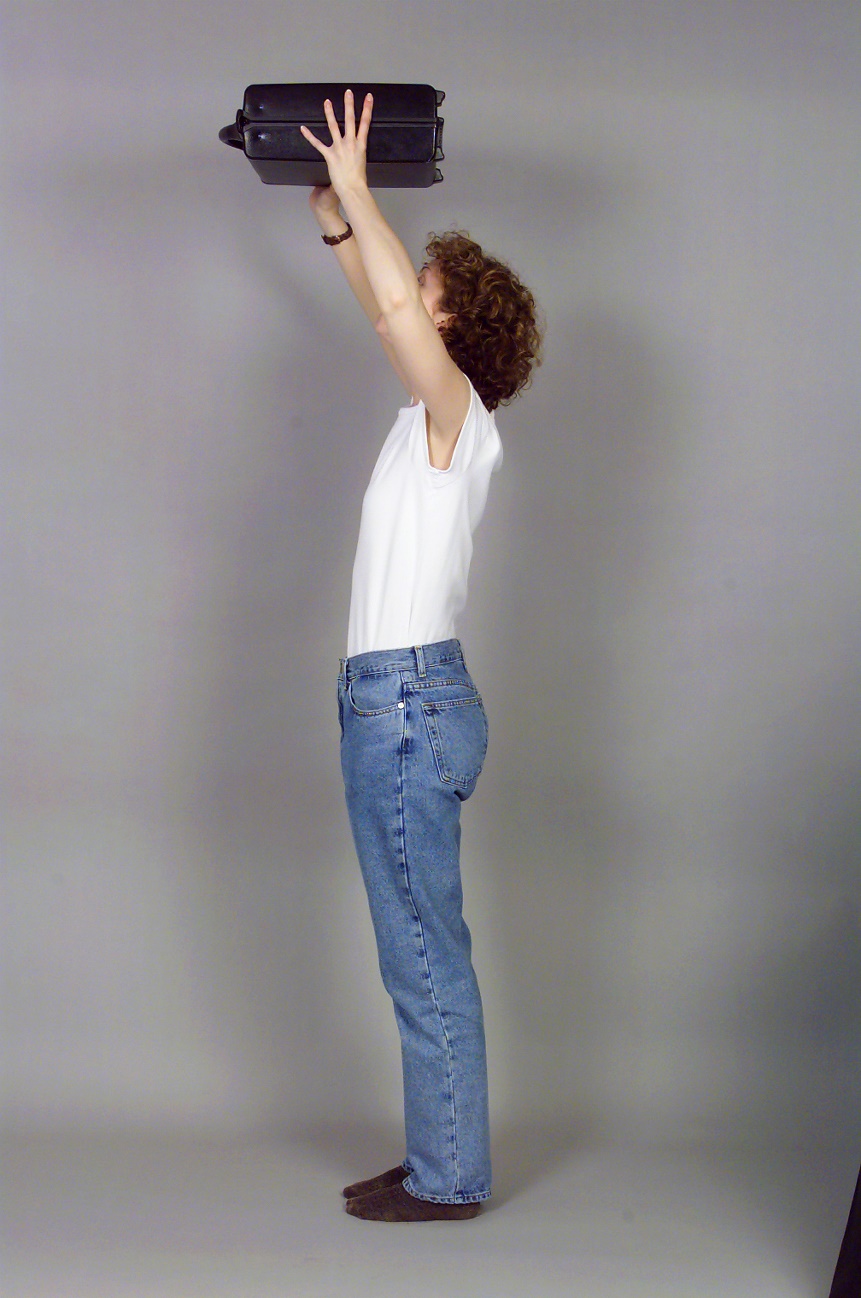  Tick one number that describes how worried or fearful of pain or injury you would be to carry out the activity shown in the picture above. | | | | | | | | | | | | | | | | |
| --- | --- | --- | --- | --- | --- | --- | --- | --- | --- | --- | --- | --- | --- | --- | --- | --- |
| No fear at all | | | |  | |  |  | |  | |  | | Extremely fearful | | | |
| 0 | 1 | 2 | 3 | | 4 | | | 5 | | 6 | | 7 | | 8 | 9 | 10 |

| 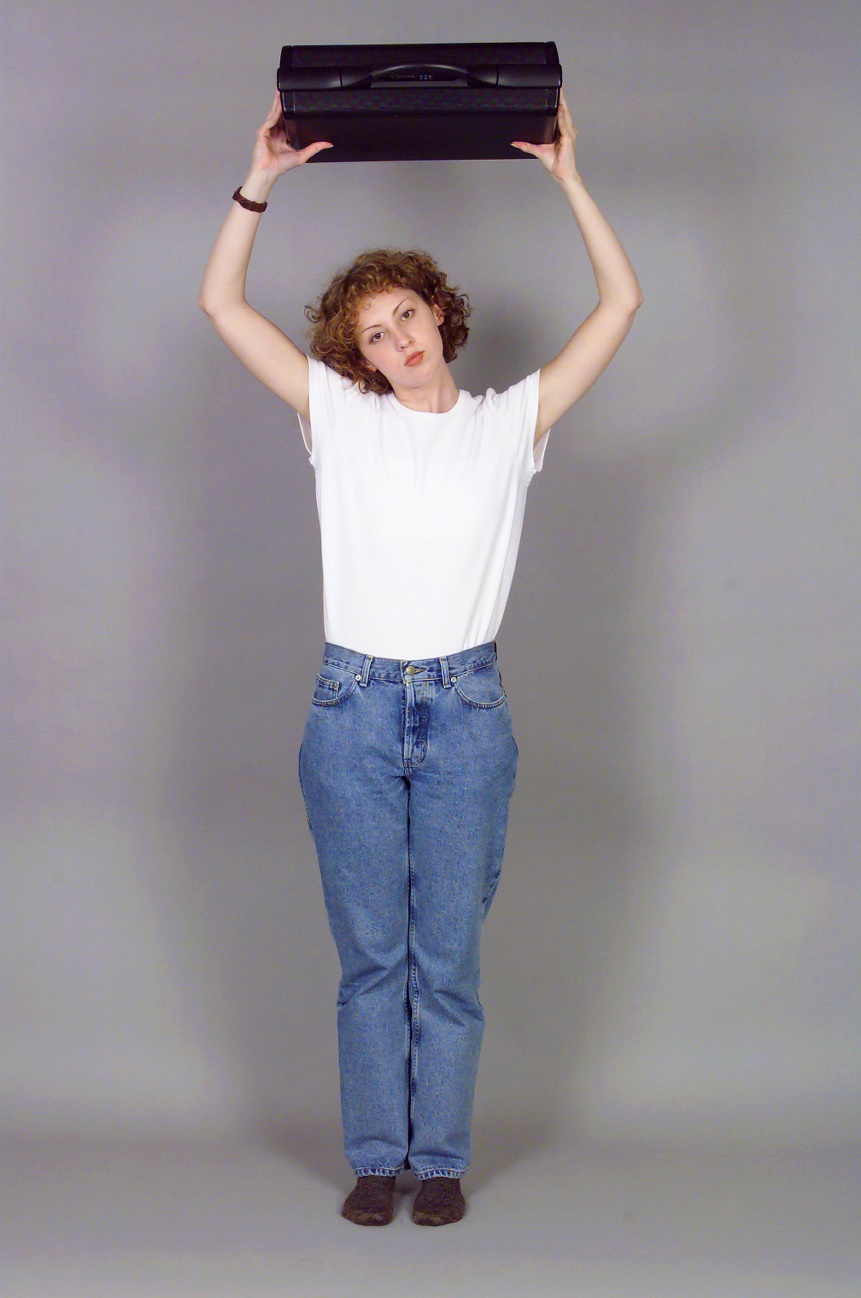  Tick one number that describes how worried or fearful of pain or injury you would be to carry out the activity shown in the picture above. | | | | | | | | | | | | | | | | |
| --- | --- | --- | --- | --- | --- | --- | --- | --- | --- | --- | --- | --- | --- | --- | --- | --- |
| No fear at all | | | |  | |  |  | |  | |  | | Extremely fearful | | | |
| 0 | 1 | 2 | 3 | | 4 | | | 5 | | 6 | | 7 | | 8 | 9 | 10 |

| 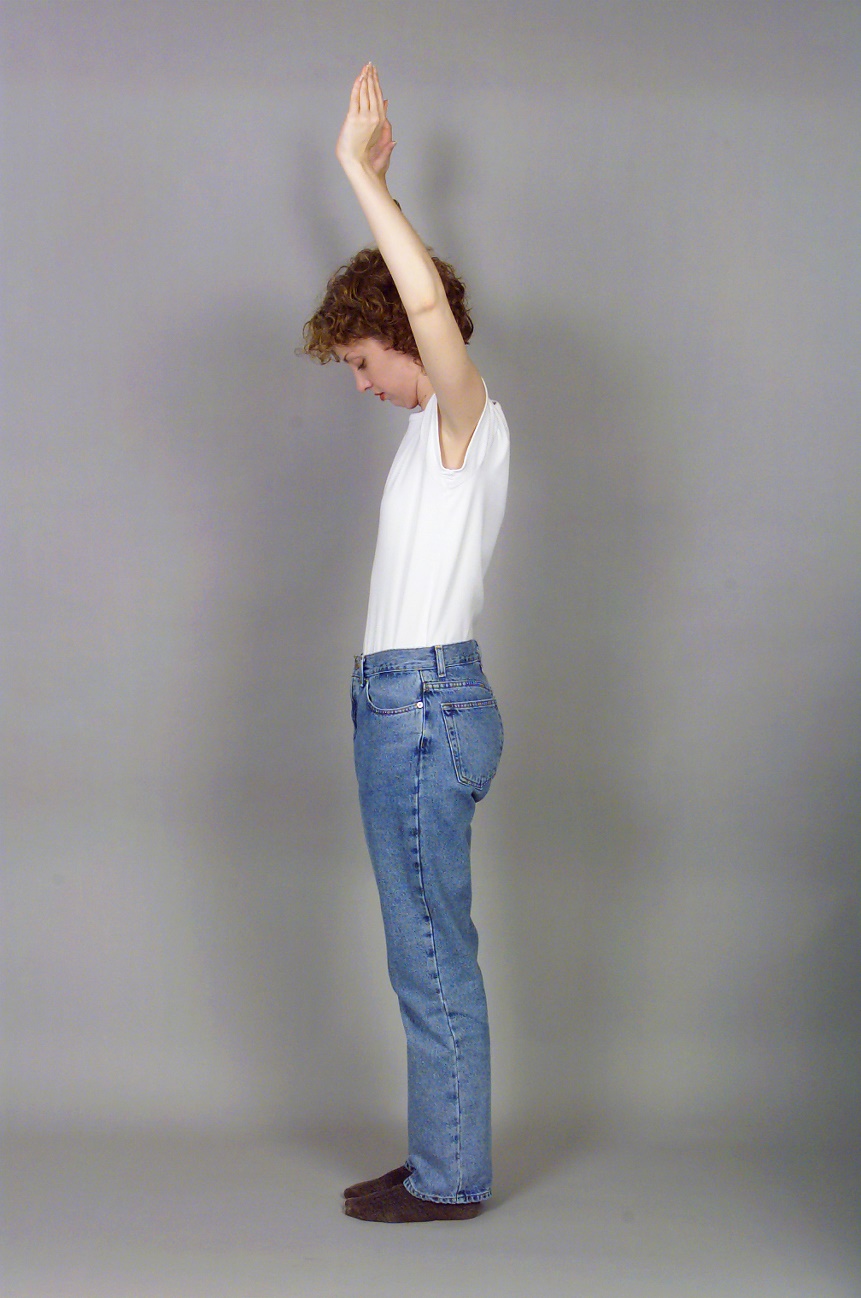  Tick one number that describes how worried or fearful of pain or injury you would be to carry out the activity shown in the picture above. | | | | | | | | | | | | | | | | |
| --- | --- | --- | --- | --- | --- | --- | --- | --- | --- | --- | --- | --- | --- | --- | --- | --- |
| No fear at all | | | |  | |  |  | |  | |  | | Extremely fearful | | | |
| 0 | 1 | 2 | 3 | | 4 | | | 5 | | 6 | | 7 | | 8 | 9 | 10 |

**Part 8. Background**

| What is your age? | | |
| --- | --- | --- |
|  | | |
| What is your gender? (Please tick) | | |
|  | Male | |
|  | Female | |
|  | Other | |
| What year were you diagnosed with Parkinson’s disease? | | |
|  | | |
| Are you on medication for treating your Parkinson’s disease symptoms? | | |
|  | No | |
|  | Yes | |
| Who do you live with? | | |
|  | On your own | |
|  | Spouse or partner | |
|  | Carer | |
|  | Other family | |
|  | Aged Care Residence or Facility | |
|  | Other | |
| Please rate the severity of your Parkinson’s disease. (If you are on Parkinson’s medications, please rate yourself based on when your medications are having a good effect). | | |
|  | No Parkinson’s disease symptoms | |
|  | Parkinson’s disease symptoms on one side only | |
|  | Parkinson’s disease symptoms on both sides, with no loss of balance | |
|  | Parkinson’s disease symptoms on both sides, physically independent but may lose balance easily | |
|  | Severe disability, but still able to walk or stand unassisted | |
|  | Wheelchair bound or bedridden unless aided. | |
| What is the postcode you currently reside in? | | |
|  | | |
| Please estimate your approximate yearly household income before tax | | |
|  | Up to $35,000 (up to $673 per week) | |
|  | $35,000- $65,000 ($673 - $1250 per week) | |
|  | $65,001- $95,000 ($1251 - $1826 per week) | |
|  | $95,001 - $125,000 ($1827 - $2404 per week | |
|  | $125,001 - $150,000 ($2404 - $2885 per week) | |
|  | >$150,000 (more than $2885 per week) | |
|  | Prefer not to answer | |
| Please select the one option that best describes your employment situation at the moment | | |
|  | Working full time | |
|  | Working part time | |
|  | Retired – pensioner | |
|  | Retired – self funded retiree | |
|  | Unemployed currently | |
|  | Home Duties | |
| In the past week, on how many days have you done a total of 30 minutes or more of physical activity, which was enough to raise your breathing rate? This may include sport, exercise, some housework (e.g. washing the car, yard and garden work, mopping and vacuuming) and brisk walking or cycling for recreation or to get to and from places but should not include physical activity that may be part of your job” | | |
|  | | |
| What effect does physical activity and/or exercise have on your pain? (please tick) | | |
|  | Completely relieves pain | |
|  | Moderate reduction in pain | |
|  | Small reduction in pain | |
|  | No change | |
|  | Small increase in pain | |
|  | Moderate increase in pain | |
|  | Large increase in pain | |
| Do you regularly use treatments and/or medications for your pain? | | |
|  | NO | |
|  | YES | |
| If yes, please list the treatments and/or medication(s): | | |
|  | | |
| If yes, in the ***last week***, how much relief have pain treatments or medications provided? Please circle the one percentage that best shows how much relief you have received. | | |
|  | 0% | No relief  Complete relief |
|  | 10% |  |
|  | 20% |  |
|  | 30% |  |
|  | 40% |  |
|  | 50% |  |
|  | 60% |  |
|  | 70% |  |
|  | 80% |  |
|  | 90% |  |
|  | 100% |  |

**Final questions**

| We are also conducting online interviews (via Zoom) which will involve answering questions about your experiences with pain, pain management techniques and exercise/physical activity. The maximum anticipated interview duration is 1 hour. Do you want to be contacted about participating in the interview?  Please be aware that if you select yes then your survey results will no longer be anonymous. | |
| --- | --- |
|  | Yes |
|  | No |
| If yes, please provide your name, email and phone number | |
| Name:  Email: Phone number: | |

Thank you for completing our survey. If you would like to opt in to be contacted about future studies OR receive a summary of the findings, please click on the link below.

**Separate survey**

Thank you for participating in our study and for your interest in either opting in to be contacted about future studies or receiving a summary of the findings. Please indicate your specific interest below:

| Do you consent to being contacted about further studies | |
| --- | --- |
|  | Yes |
|  | No |
| Would you like to receive information about the overall results of this study? | |
|  | Yes |
|  | No |
| If yes (to either question), please provide your name, email and/or postal address | |
| Name:  Email:  OR  Postal address: | |
