# Supplementary material for: A Mixed Methods Approach to Explore the Experience of Pain and Its Management in People with Parkinson's Disease
Source: Parkinsons Dis. 2024 May 25;2024:8515400. doi: 10.1155/2024/8515400 (PMC11144069; doi:10.1155/2024/8515400)
Supplement: Supplementary Materials — Supplementary material provided includes: Supplementary Figure: Study design, Survey, Supplementary Table 1: Descriptions of measurements (expanded version), Interview guide, and Supplementary Table 2: Supporting quotes. [file 8515400.f1.zip › c. PD_Supplementary_Table 1_v1_amended_quality check.docx]

**Supplementary table 1. Description of measurements (expanded version)**

| Measurement tools used | Description | Categorization for pain contributors |
| --- | --- | --- |
| Global Pain Measures:  The category(s) of pain present, along with the severity of pain and it’s level of interference on daily activities. | | |
| The King’s PD Pain questionnaire* | - Time frame: past month - Identifies categories of pain - 14 item scale, consisting of 7 domains:  1. Musculoskeletal 2. Chronic 3. Fluctuation related 4. Nocturnal 5. Oro-facial 6. Discoloration and edema/swelling 7. Radicular pain  - Score range: 0 - 14 | N/A; collected to assist description of pain categories |
| Brief Pain Inventory – severity and interference subscores* | - Time frame: past week - 8 item questionnaire - Pain severity and interference sub-scores - Sub score range: 0 - 10 | <2 = very low ≥2 - <4 = low ≥4 - <7 = moderate ≥7 - <9 = high ≥9 - 10 = very high |
| Peripheral Neuropathic contributor:  Pain caused by a lesion or disease of the peripheral nervous system | | |
| Self-reported Leeds Assessment of Neuropathic Symptoms and Signs pain scale (S-LANNS)* | - Time frame: past week - 7 item scale - A score ≥ 12 suggests pain of predominantly neuropathic origin - Score range: 0 - 24 | <5 = very low ≥5 - <10 = low ≥10 - <15 = moderate  ≥15 - <20 = high  ≥20 - 24 = very high |
| Central nociplastic contributor:  Pain that arises from altered nociception despite no clear evidence of actual or threatened tissue damage causing the activation of peripheral nociceptors or evidence for disease or lesion of the somatosensory system causing the pain | | |
| Central Sensitization Inventory (CSI)* | - Time frame: time period not specified - 25 item assessment - A score ≥ 40 is suggestive of central sensitization syndrome - Score range: 0 - 100 | 0 - 20 = very low ≥20 - <40 = low ≥40 - <60 = moderate ≥60 - <80 = high ≥80 - 100 = very high |
| Emotional dysregulation or pathology contributor:  Impaired ability to regulate and or tolerate negative emotional states, including anxiety. | | |
| Parkinson's Anxiety Scale (PAS)* | - Time frame: past month - 12 item scale with three subscales, for persistent, episodic anxiety and avoidance behavior. - A score of 13/14 is suggestive of patients with anxiety disorder - Score range: 0 - 48 | 0 - <6 = very low ≥6 - <13 = low  ≥13 - <24 = moderate  ≥24 - <36 = high ≥36 - 48 = very high |
| Maladaptive cognitions contributor:  Inaccurate or irrational beliefs, thoughts or behaviors about, or resulting from, the experience of pain. | | |
| Brief Pain Catastrophizing Scale (PCS)* | - Time frame: time period not specified - 4 item scale - A score of 6 and 9 is suggestive of a moderate and high level of catastrophizing. - Score range: 0 - 16 | 0-2 = very low 3-5 = low 6-8 = moderate 9-12 = high  13-16 = very high |
| Pictorial Fear of Activity Scale (PFACTS)* | - Time frame: time period not specified - Instrument with 19 images of a model engaged in a variety of activities. Participants were asked to view each picture and indicate “how worried or fearful you would be to carry out the activity shown in the picture” on a scale from 0 (“no fear at all”) to 10 (“extremely fearful”). - Score range: 0 - 190 | 0-38 = very low  39-76 = low  77-114 = moderate 115-152 = high 153-190= very high |
| Socioenvironmental contributor:  Socioeconomic factors (eg income and employment status) which can interact with physical pathology to modulate a patient’s report of symptoms and may worsen the clinical presentation but also access to appropriate care. | | |
| Median income ($AU) | - Time frame: Yearly household income assessed before tax - 0 = Up to $35,000 (up to $673 per week)  1 = $35,000- $65,000 ($673 - $1250 per week)  2 = $65,001- $95,000 ($1251 - $1826 per week)  3 = $95,001 - $125,000 ($1827 - $2404 per week  4 = $125,001 - $150,000 ($2404 - $2885 per week)  5 = >$150,000 (more than $2885 per week)  6 = rather not say | 5 = very low  4 = low  3 = moderate  2 = moderate – high  1 = high  0 = very high  6 = N/A |
| Employment status | - Time frame: current employment status - Employment status included: Home duties, retired (self-funded), retired (pensioners), currently unemployed, working part time, working full time. | N/A, collected to aid in interpretation of median income data. |

*high score is worse
