# Supplementary material for: A Mixed Methods Approach to Explore the Experience of Pain and Its Management in People with Parkinson's Disease
Source: Parkinsons Dis. 2024 May 25;2024:8515400. doi: 10.1155/2024/8515400 (PMC11144069; doi:10.1155/2024/8515400)
Supplement: Supplementary Materials — Supplementary material provided includes: Supplementary Figure: Study design, Survey, Supplementary Table 1: Descriptions of measurements (expanded version), Interview guide, and Supplementary Table 2: Supporting quotes. [file 8515400.f1.zip › d. PD_Supplementary_Interview guide_V1_quality check.docx]

**Supplementary material – Interview guide**

**Exploring pain management techniques and the relationship between pain and physical activity/exercise in people with Parkinson’s disease: Interview guide**

**Aim of the interviews:** To explore the similarities and differences in a) pain management techniques and b) the relationship between pain and physical activity/exercise amongst individuals with different pain ‘contributors’.

**Script for Oral Consent**

I have received your signed consent form; do you have any questions about this consent form, participant information statement or study? With your permission, I would like to make an audio recording of our discussion to make sure I am getting an accurate record of your thoughts today. Do you give me permission to audio record our interview today?

**Topic Guide**

| **Topic** | **Aim** | **Proposed questions** |
| --- | --- | --- |
| **Impact of pain** | **To explore the impact of pain on the lives of people with Parkinson’s disease** | As you know we are investigating the different experiences that people with Parkinson’s disease have with pain. Your questionnaire responses have given me some picture of the way your pain behaves; however, I am interested in hearing now about your personal experience. We know living with chronic pain impacts people’s lives in different ways. It could be in a physical way, a psychological way or in their social lives. With that in mind, I would like you describe the impact pain has had on your life.  *Prompts:*   - *What is it like to live with pain if you were to explain it to someone else?* - *How does the pain affect the activities you do?* - *How does the pain affect how you feel emotionally?* - *How does the pain affect your social activities?* |
| **Pain management** | **To understand how the pain management techniques used by the participant have evolved over the course of their current pain condition.** | I am interested in what you do to manage or treat your pain. Thinking about strategies you do for yourself i.e. self-help strategies to manage pain, could you describe for me what you currently use to manage your pain and how this is similar or different to things you have used in the past.  *Prompts:*  *How has your choice in pain management techniques changed/evolved over the years, if at all?*  How have these <mentioned pain management techniques> influenced, if at all, your ability to cope with your pain?  What has been your experience with different health professionals you have seen in managing your pain over the years?  Prompts:  *Who in healthcare do you turn to for help/advice about your pain?*  *In what ways, if any, do these health professionals make a difference to your pain?* |
| **Relationship between exercise/physical activity and pain.** | **To understand the relationship between exercise/physical activity and pain in people with Parkinson’s disease.** | 1. We are going to talk about exercise and physical activity and pain. When you hear the word exercise, what sort of things come to mind? What comes to mind when you hear the words physical activity? 2. We know different people experience different responses to exercise and physical activity, with some reporting their pain gets better and others worse. What influence, if any, does physical activity and exercise have on your pain?   *Prompts:*   - - *Are there particular types of exercises (i.e. strength, aerobic, water-based etc) and activities you would/wouldn’t recommend someone with Parkinson’s disease in pain to try given your experience?*  1. Thinking about your usual exercise regime, if any. Has your decision to exercise or not exercise been influenced by your pain? If so, in what ways?   *Prompts:*   - - *Are exercise and physical activities something you would recommend/or not for pain management? Please explain why?*  1. What activities, if any, have you altered as a result of your pain and how did you come to this decision? (This could be changing activities, stopping some or starting new ones)    - Please explain the impact of this decision |
| That’s all of my formal questions. Is there anything that you wanted to add? | | |
