# Supplementary material for: A Mixed Methods Approach to Explore the Experience of Pain and Its Management in People with Parkinson's Disease
Source: Parkinsons Dis. 2024 May 25;2024:8515400. doi: 10.1155/2024/8515400 (PMC11144069; doi:10.1155/2024/8515400)
Supplement: Supplementary Materials — Supplementary material provided includes: Supplementary Figure: Study design, Survey, Supplementary Table 1: Descriptions of measurements (expanded version), Interview guide, and Supplementary Table 2: Supporting quotes. [file 8515400.f1.zip › e. PD_Supplementary_Table 2_V1.docx]

**Supplementary table 2. Supporting quotes**

| **Theme** | **Supporting quotes** | | | |
| --- | --- | --- | --- | --- |
| **Causative perceptions of pain are diverse** | Causative perceptions – motor impairments: *“It’s developed through the Parkinson’s…I've got two types of pain: one is in my hip flexor…which I think is due to the rigidity which is worst in that area, and…the other pain is the dystonia” (P21,PD-22yrs).*  Causative perceptions – lack of diagnostic certainty: *"I think it's very difficult for people to sort of understand what pain is like with Parkinson's and I guess it exacerbates…any other pain you might’ve had that’s not Parkinson’s related" (P7,PD-16yrs).*  Causative perceptions (as informed by others) – independent of PD: *“that’s just what everybody says, “oh it’s just old age, you get stiff around the shoulders” and the doctors told me “wear and tear”” (P17,PD-14yrs).* | | | |
| **Sense of control** |  | Participants communicating a higher sense of control | Participants communicating a l*ower sense of control* | Participants communicating less extreme senses of control |
|  | Level of acceptance | *“It's a difficult thing to get control of, to understand, and it's taken me a long time to accept the fact that that pain is going to be there forever”* *(P20,PD-3yrs).* | *“Pain is my world. I can't do I can’t act and do what I used to do…I can't do anything I can't plan for anything”* and “*I was extremely successful…I lost everything because of it* [pain]*, it's terrible*.” *(P11,PD-12yrs)* | “*It still really hasn't sunk in that what doesn't get done today will get done tomorrow. I was always on a deadline in my work, and you have to get it done and I don't think I've got rid of that completely yet”* *(P15, PD 1 year)* |
|  | Coping | *“I just try and live with the pain and I don’t like it to take over my life”* *(P6,PD-5yrs).* | “*Walking up the hill stopped altogether and it wasn't a decision; it just wasn’t possible…it was just the pain that stopped me from doing it….it’s not an active decision of mine*” *(P9,PD-3yrs)* | *“I'm learning that if I do too much well then that that knocks me about too much the next day and I can't do anything so I've gotta learn to balance it all out and do a little bit of everything” (P16, PD 1 year).* |
|  | Physical activity | “*I've had to learn to temper myself if I stop doing an activity which is too painful” (P6,PD-5yrs)* and “*If I'm going to do something I still do it even if I'm in a little bit of pain it just impacts how I do them …it doesn't stop me from doing things it just means that when I do do things they are a little bit more complex and … they take longer*.” *(P22,PD-2yrs)* | “*I’m just so tired I just feel, so no energy to do them and then I just walk away from them, then I think feel guilty, go and do some exercise. You know it’s a fight between the fatigue and my knowledge that they are good and beneficial*” *(P14,PD-17yrs) and* “*cause if I’m in pain I don’t probably feel like doing exercise*” *(P24,PD-6yrs)* | *“It's just that context that means you go each week, and you think I can do this, is this getting better or am I letting things slip so there’s a sort of monitoring aspect to it as well I think cause you're in a different setting” (P12, PD 1 year)* |
| **Belief in the value of therapy** | Lack of long-lasting solution: “*I used tablets for cramps then I’ve tried deep heat rubs and that on the back of my leg, that probably had a better effect than the tablets, but you know it's only lasting about 3-4 hours type of thing.*” *(P5,PD-5yrs)*  Belief that exercise would reduce their pain: *“I realised there was a link, and the more body strength I get back the better it gets, and the more mobility I get the better it gets and the better your pain gets” (P9,PD-3yrs) and* *“Of all my pills, boxing [exercise] is the best one for me.”* *(P18,PD-17yrs)* | | | |
| **Pain is the unspoken symptom of Parkinson’s disease** | Social withdrawal: *“It often stops me from going out or participating in things because I'm in so much pain or discomfort”* *(P21,PD-22yrs) and “It stops socially because you kind of get cranky, you know what I mean, when you're in pain you don't want to be around people”*. *(P12,PD-1yr)*  Pain not being addressed by healthcare providers: “*I think it’s really difficult for physicians to understand what pain can mean for Parkinson’s”* *(P7,PD-16yrs)*  Allied health professionals reactive approach to pain: *“Physio appointments have been about maintaining capabilities that could degrade because of the Parkinson's so fine motor skills those sort of things not pain related…certainly with the formal exercise sessions where I've got a sore knee today and was given different exercises at those times to do” (P3,PD-2years)* | | | |
